# Removal of *E. coli* O157:H7 coliform bacteria from sewage wastewater using silver doped borate bioglass

**DOI:** 10.1038/s41598-025-11844-8

**Published:** 2025-07-26

**Authors:** Bassant I. Elsaba, Ashraf Elsayed, Yasser S. Rammah, Hosam Salaheldin

**Affiliations:** 1 Botany Department, Faculty of Science, Mansoura University, Mansoura, 35516 Egypt; 2 Department of Physics, Faculty of Science, Menoufia University, Shebin El-Koom, Menoufia 32511 Egypt; 3 Biophysics Research Group, Physics Department, Faculty of Science, Mansoura University, Mansoura, 35516 Egypt

**Keywords:** Wastewater treatment, Borate BG, Silver ions, *E. coli* O157:H7, Biological techniques, Biophysics, Microbiology

## Abstract

The depletion of freshwater resources constitutes a significant global issue, prompting the consideration of treated sewage wastewater (SWW) reuse as a viable alternative water source within contemporary water management strategies. The current study represents an innovative silver borate-based bioglass (BG) system for the treatment of municipal SWW. The melting-quenching method was used to prepare silver doped BG samples with different Ag_2_O concentrations. BAg1, BAg2, BAg3, and BAg4 BG samples were obtained from (65-X) B_2_O_3_–20Na_2_O–10CaO–5P_2_O_5_–XAg_2_O (where X = 0, 1, 2, 3, and 4 mol%). A major fecal coliform bacterium, *Escherichia coli* O157:H7 (*E. coli* O157:H7), was used as an indication of SWW purification. BG samples were characterized using FTIR, XRD, SEM, EDX, and DLS, respectively. BAg1 BG sample showed the strongest antibacterial activity against *E. coli* O157:H7, with a 27.0 ± 1.0 mm inhibition zone and 95.31% growth inhibition. Compared to other BG samples, the inhibition zone diameter was marginally lower than the amoxicillin positive control (28.33 ± 0.1 mm). Additionally, all BG samples inhibit the coliform bacterial community approximately 100%., whereas the original BG sample (BAg0) did not inhibit coliform bacteria. All BG samples had minimum inhibition concentrations (MICs) of 5 mg/mL and minimum bactericidal concentrations (MBCs) of 50 mg/mL, respectively, compared to the untreated bacterial culture (− Ve control). SEM micrographs showed that BAg1 sample treated *E. coli* O157:H7 cells deteriorated more than BAg0. In conclusion, silver-doped BG is a cost-effective, eco-friendly, and highly recommended technology for SWW purification from fecal coliform bacteria and therefore, can be used for reclamation and other non-drinking water.

**Supplementary Information:**

The online version contains supplementary material available at 10.1038/s41598-025-11844-8.

## Introduction

Almost 70% of our globe is made up of water, with a total volume of nearly 1400 million Km^3^ Liu, X^1,2^, of which freshwater makes up around 3%. Of this, just 0.01% is suitable for human use^3^. The previous few decades have seen a sharp rise in the demand for freshwater as a result of industrialization, urbanization, and population growth^4^. Sewage wastewater, or the continuous contamination of freshwater resources by microbes and organic and inorganic chemicals, is the main problem in the water supply chain^5^.

Wastewater is often defined as water that has been tainted by human activities^6^. Along with feces and urine, it also contains a variety of substances that are dumped into the sewer after usage, including detergents, medications, and cosmetics. About 99.9% of wastewater is made up of water^7^, as well as organic and inorganic materials like phosphorus, nitrogen, sulphates, chlorides, and bicarbonates, as well as microbes, suspended solids, and hazardous substances^8^. Depending on whether it is classified as residential, commercial, industrial, or a combination of these, the contents impart unique physical, chemical, and biological qualities^9^.

In poor countries, 90% of illnesses are caused by drinking tainted water^10,11^. Wastewater is home to several billions of fecal microorganisms. These bacteria can’t survive for long periods outside of their "extra-intestinal" environment, which is their natural habitat. In the nutrient-rich aquatic environment of the microbiological sewage wastewater treatment reactor, numerous saprophytic bacteria outcompete them. Sewage effluent’s organic and inorganic components give these “saprophytes” nourishment, energy, and cell carbon. They are quite well adapted to this environment, in contrast to fecal bacteria, which either die quickly or slowly^12^. The most isolated saprophytic bacteria in aerobic microbiological treatment systems are facultative, Gram-negative, and heterotrophic rods. *Achromobacter, Bacillus, Flavobacterium, Pseudomonas, Zooglea*, and non-fecal coliforms are among the Proteobacteria that belong to these genera^13^.

Sewage wastewater (SWW) treatment can be classified based on the type of treatment process used, such as chemical, physical, or biological methods^14,15^. Wastewater is biochemically broken down into stable end products by microbes, mostly bacteria, in biological treatment processes. A portion of the trash is converted into carbon dioxide, water, and other byproducts, and a larger number of microorganisms, or sludge, are produced^16,17^. Sedimentation (Clarification), screening, aeration filtration, flotation, skimming, degasification, and equalization processes are examples of physical treatment methods, which use only physical phenomena to improve or treat wastewater without requiring major chemical or biological changes^18^. Chemical treatment is the process of improving the quality of water by using particular chemical processes. Chlorination is the chemical method that is commonly used. The powerful oxidizing agent chlorine is used to get rid of bacteria and slow down the rate at which wastewater breaks down. As an oxidizing disinfectant, ozone, another strong oxidizing agent, has been used. The chemical process of neutralization is widely used in many industrial wastewater treatment operations^18,19^.

The elimination effectiveness of bacterial and viral pathogens from sewage wastewater treatment systems should be comparable to that of fecal pathogens. Its numbers should be easy to count and cost-effective, while ensuring accuracy and dependability^20–22^. Although no bacterium regularly meets all of these requirements, one particular coliform bacterium, *E. coli*, comes close. *E. coli* is the main species in the fecal coliform group. Among coliform bacteria, *E. coli* is the most accurate indicator of fecal contamination and possible pathogen presence because it is normally absent from environmental development and reproduction^23^.

However, some *E. coli* strains, especially O157:H7, can cause serious illness. *E. coli* O157:H7 in surface waters represents a risk to human health through drinking or ingestion during recreational activities^24^. The zoonotic life-threatening Shiga toxin-producing *E. coli* O157:H7 (STEC) was first described in 1982 and has since become a significant food- and water-borne pathogen that causes hemolytic uremic syndrome, hemorrhagic colitis, and diarrhea in humans^25^.

Larry Hench created 45S5 bioglass®, a biocompatible glass consisting of 45 SiO_2_, 24.5 Na_2_O, 24.5 CaO, and 6 P_2_O_5_ (wt.%), in 1969 to treat hard tissue injuries such as bone fractures. To increase biocompatibility, synthetic materials adhere to both soft and hard tissues and encourage the growth of new blood vessels^26,27^. A variety of BG compositions have been developed to address non-skeletal applications^28^. Several BG formulations based on borate have been created to treat soft tissue injuries, either with or without biopolymers^29^. Different quantities of additional oxides are integrated into silicate, borate, or phosphate bioactive glasses to bestow certain features to the material; for example, CaO, K_2_O, Na_2_O, and MgO are beneficial for modifying the surface^30^. Reactivity within the biological environment; ZnO, CuO, and Ag_2_O facilitate the release of biologically active ions possessing antibacterial characteristics^30^. Up to the best of our knowledge, limited studies were made around the use of borate BG doped with silver and actually applied for wastewater and/or SWW coliform bacterial removal. While the use of BG doped with or without silver doping were investigated in-vitro^31–33^.

Ying Xu et al. (2008) examined the antibacterial efficacy of borosilicate glass containing 0%, 1%, 1.5%, 2%, and 3% Ag_2_O against *E. coli*, observing a noticeable antibacterial zone that expanded in size from 1 to 6 mm with increasing Ag_2_O concentration^34^. A melt-quenched bioactive glass from the SiO_2_-CaO-P_2_O_5_-MgO system was changed by incorporating 1 and 2 mol% Ag_2_O, as well as glasses containing 1 mol% Ag_2_O. The antibacterial efficacy of the glasses against *E. coli* was often enhanced by reducing particle size or raising Ag_2_O concentration^33^. Thin films of silver and samarium-doped BG were effectively manufactured using pulsed laser deposition (PLD) and spin coating, resulting in significant antibacterial activity, with inhibition zones of 2.33 mm against *E. coli* and 3.33 mm against *B. subtilis*^35^. Therefore, the obtained purified SWW can be used for laundering, vehicle washing, watering of gardens, crops, and lawns, as well as certain industrial processes. Luo et al., (2010) reported that borate glasses with the composition 54B_2_O_3_-22CaO-6Na_2_O-8K_2_O-8MgO-2P_2_O_5_ (mol%) doped with 0.75, 1, and 2 wt% Ag_2_O^36^. In vitro tests on osteoblasts and fibroblasts revealed that those doped with 2 wt% Ag_2_O were toxic, but those doped with 0.75 and 1 wt%Ag_2_O showed no harmful effects^36^.

The aim of this work was to develop and evaluate a borate-based bioglass (BAgX) system doped with varying concentrations of silver oxide (Ag_2_O) for the effective purification of sewage wastewater (SWW) contaminated with fecal coliform bacteria, specifically *E.coli* O157:H7, a key indicator of fecal contamination. Confirmation tests for *E. coli* growth in EC broth and on TBX agar media were examined. Silver ion release from Ag-doped BG in both SWW and deionized water was investigated. The antibacterial activity test, minimum inhibitory concentration (MIC) assay, and minimum bactericidal concentration (MBC) assay were made. Additionally, detection of Ag-doped borate BG samples—resistant variants test was conducted. The morphological structure of *E.coli* O157:H7 cell membrane deterioration using SEM imaging was investigated to estimate the efficiency of the Ag-doped borate BG system. Therefore, this cost-effective, and simple BG system is highly recommended for several non-drinking water uses such as irrigation, industrial processes, toilet flushing, laundry, and vehicle washing.

## Materials and methods

### Chemicals

The composition of the different types of BG (BAgX) samples is depicted in Table 1. The following chemicals NH_4_H_2_PO_4_ in (99.90%, Alfa Aeser), H_3_BO_3_ in (99.90%, Alfa Aeser), CaCO_3_ in (99.90%, Alfa Aeser), and Na_2_CO_3_ in (99.90%, Alfa Aeser) were used as precursors of metal oxides P_2_O_5_, B_2_O_3_, CaO, and Na_2_O, respectively. Additionally, Ag_2_O (99.90%, Alfa Aeser) was added with different concentrations (AgX = 1, 2, 3, and 4 mol %). Table. 1 depicts the composition of the prepared silver-doped borate-based BG samples.

**Table 1:** Composition of the prepared silver-doped borate-based BG (mol%) samples (BAg1, BAg2, BAg3, and BAg4).

Oxides	Composition (mol%) of Ag doped BG samples				
	BAg0	BAg1	BAg2	BAg3	BAg4
B_2_O_3_	65	64	63	62	61
Na_2_O	20	20	20	20	20
CaO	10	10	10	10	10
P_2_O_5_	5	5	5	5	5
Ag_2_O	0	1	2	3	4

### Sewage wastewater (SWW) samples

Samples of SWW were obtained randomly from the Mansoura wastewater treatment facility. Samples were conveyed to the Bacteriology laboratory under the Botany Department at the Faculty of Science, Mansoura University, Egypt. The pH of the samples was subsequently measured using an Adwa Benchtop Meter AD1200 (Hungary) and preserved in a White Whale No Frost Freezer 5 Drawers WF-205NKS Model (Egypt) at 4 ℃.

### Bacterial isolate

*E. coli* O157:H7(CP008957) as a core of water-borne pathogens that cause serious diseases was obtained from the gene bank (Cairo, Egypt). *E. coli* O157:H7 was grown on Luria–Bertani (LB) medium by mixing these components in one distilled liter of water that are 10.0 g sodium chloride (NaCl, 99.90%, Piochem, Egypt), 10.0 g peptone and 20.0 g agar (99.90%, Lanxess, India), 5.0 g yeast extract (99.90%, Piochem, Egypt), at pH = 7.0.

### Preparation of BAgX BG samples

**Fig. 1:**
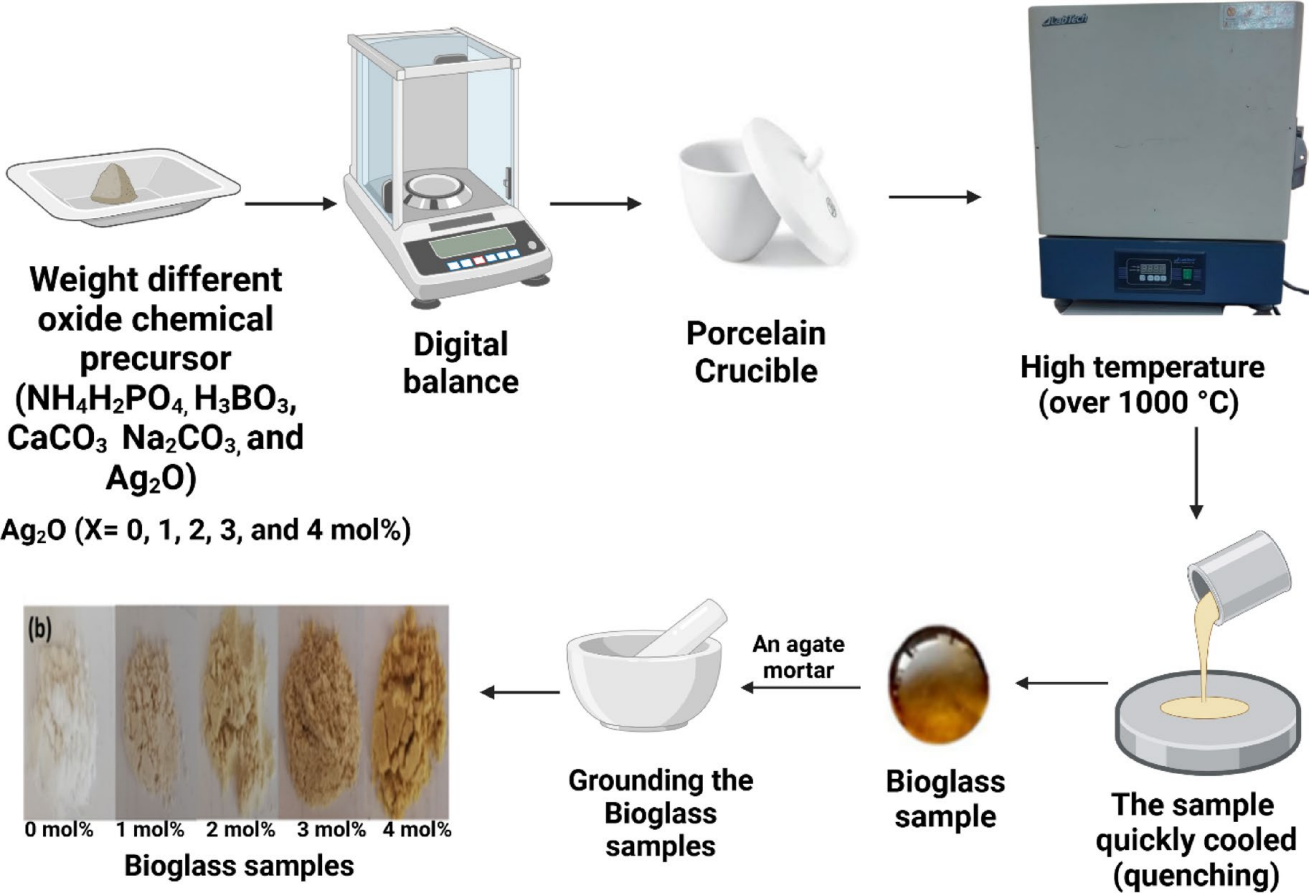
The schematic diagram of the preparation procedure of the BAgX bioglass samples.

The BG samples were made using the melt-quenching approach, which produces an amorphous BG sample by melting precursor oxide powders at high temperatures (over 1000 °C) and then quickly cooling (quenching) the melt. An agitated mortar was then used to grind the samples, and the resulting BG powder samples were refined (Fig. 1). To get a fine powder with an average diameter of roughly 10 µm (μm), the powder was lastly refined using a specialised sieve^37^. During the melt annealing process, Si_2_O was fully substituted with B_2_O_3_ in equivalent amounts to create the BG samples^38^. The composition of BAgX was (65-X) B_2_O_3_-20Na_2_O-10CaO-5P_2_O_5_-XAg_2_O (X = 0, 1, 2, 3, and 4 mol%). The samples will be labelled as BAg1, BAg2, BAg3, and BAg4 based on the amount of Ag_2_O used instead of B_2_O_3_, ranging from 0 to 4 mol% in a platinum crucible.

In the muffle furnace (Lab Tech 1200, China), the Ag doped BG samples were melted at 1150 ± 10 °C for 30–40 min based on the sample type, and the heating rate was 4 °C /min.^39^. To achieve the required homogeneity of the molten samples, they were rotated throughout the melting process. After then, the melted samples were warmed at 400 °C in a stainless-steel mold. The muffle was kept for one hour at this temperature before, it turned off to cool to room temperature at a rate of 30 °C per hour^40^. The preparation steps of the Ag doped BG samples are represented in Fig. 1.

### Characterization techniques

#### Fourier transform infrared (FTIR) spectroscopic

FT-IR spectra measurements were carried out using a Bruker FT-IR spectrometer (Invenio S, Germany). The spectral resolution was 4 cm^−1^ in the range of 400–4000 cm^−1^. The BAgX (BAg0, BAg1, BAg2, BAg3, and BAg4) samples were uniformly mixed with solid KBr before being compressed to conduct FTIR analysis, which was carried out scanned at room temperature (25 ± 2 °C).

#### X-ray diffraction (XRD) spectroscopic

Using an x-ray diffractometer (PANalytical X’Pert Pro Multipurpose diffractometer), powder samples were subjected to Cu-Kα x-ray radiation (λ = 1.5418 Å) at 45 kV and a current of 30 mA with a step size of 0.02° and a scan speed of 1°/min. Examined was the crystalline structure of the samples.

#### Scanning electron microscopy (SEM) and energy-dispersive X-ray (EDX) spectroscopy

The JEOL JSM-6510LV (USA) scanning electron microscope equipped with an integrated energy dispersive X-ray spectroscopy (EDS) detector is typically operated at an accelerating voltage of 30 kV and a working distance of 12 mm for analysis of silver-doped BG samples.

#### Dynamic light scattering (DLS)

The average particle size of the prepared BG samples (BAg0 and BAg1) was examined using a Malvern Zeta sizer Nano series (Nano ZS90, UK) compact scattering spectrometer. The BAg0 parent and BAg1 (1.0 mg (mg)) BG sample was added to 5 mL of deionized H_2_O. Then, the two BG samples were sonicated for 20 min before measurement.

### Treatment of sewage wastewater (SWW) samples using BAgX BG samples

#### Standard plate count (SPC) method

The effect of all types of BAgX (X = 0, 1, 2, 3, and 4) BG samples on the coliform bacterial community in SWW samples was performed on MacConkey agar plates (99.90%, ready-made media from Mast Group, United Kingdom). As a positive control, the SWW sample was treated with 0.4% calcium hypochlorite (Ca (ClO)_2_, 40%). In sterilized bottles, 0.2 g of the BAgX BG samples were separately incubated in 10 mL of SWW samples at 28 ± 2 °C and 150 rpm for overnight incubation. After that, streaking 50 µl (μL) from each treated sample in addition to positive and negative controls was performed on MacConkey agar plates and incubated at 37 °C for 24 h, followed by counting the pink colonies of coliform bacteria.

To detect the presence or absence of coliform bacteria in SWW after treatments with BAgX BG samples compared to the positive control, Ca (ClO)_2_. Plates with 20–300 pink colonies are taken into consideration for counting. Furthermore, the count of colonies was expressed as colony-forming units per millilitre (CFU/mL). After that, the removal percentage of coliform bacteria in treated SWW was calculated according to Eq. (1).

1 $$\text{Removal}\mathbf{\%}=\frac{\text{No}.\text{ of colonies in untreated sample}-\text{No}.\text{ of colonies in treated sample }(\text{CFU}/\text{mL})}{\text{No}.\text{ of colonies in untreated sample }(\text{CFU}/\text{mL})}\text{x }100$$

#### Multiple-tube fermentation method

##### Presumptive test

The Most Probable Number (MPN) techniques, as previously published by Ibe and Okplenye [41], were used to determine the coliform counts of SWW samples using sterile MacConkey broth. The SWW samples were treated with all types of BAgX at a concentration of 20 mg/mL and incubated overnight at 28 ± 2 °C under shaking conditions at 150 rpm. This experiment was conducted in 3 groups, differing in the inoculum size of 5, 1, and 0.1 mL; each group consisted of 5 tubes or replicas. In the first group, the 5 mL sample from each treatment was inoculated into 5 mL MacConkey broth media (double strength). In the second group, one mL of each treated sample was added to a tube containing 5 mL of MacConkey broth (single strength). In the third group, 0.1 mL from each treated sample was added to a tube containing 5 mL of MacConkey broth (single strength), and SWW samples without BAgX treatment were used as control. All tubes were incubated at 37 °C for 24–48 h.

After 24 and 48 h of incubation, each tube was examined for acid and gas production. The colour of the media changes from purple to yellow, and on the collection of gas in Durham’s tube, it can be assumed that coliform bacteria are present in these samples. The number of combinations of positives was counted from each set after 48 h and the MPN value from the standard chart (**Table. S1**) of the MPN index was calculated as MPN/100 mL according to Eq. (2).

2 $$\text{MPN}/100\text{mL }=\left(\text{MPN index}/100\text{ mL}\right)\times (10/\text{V})$$

where V = volume of one sample portion at the lowest selected dilution to give a coliform count per 100 mL of water sample (MPN/100 mL).

##### Confirmation test for the detection of *E. coli* in EC broth and on TBX agar media

EC broth medium (EC, 99.90%, ready-made media from Titan Biotech, India), a selective medium for *E. coli,* was used to differentiate *E. coli* that ferments lactose at 44.5 °C from Gram-negative coliform bacteria^42^. In the confirmation test, all positively presumptive test tubes (yellow colour and producing gas) from the presumptive test were subculture in EC broth media to determine if the coliform bacterial mixture included *E. coli* as fecal indicator bacteria.

This experiment was conducted in three groups, and each group consisted of 5 tubes containing 10 mL of EC broth media. A 0.1 mL of each positive tube in the presumptive test was injected into each corresponding tube containing EC broth media. The final pH was adjusted at a value of 6.9 ± 0.2 (at 25 °C) in fermentation tubes containing the inverted Durham’s tube that was used to detect the gas formation. The number of positive tubes (with turbidity and gas production) observed compared to the untreated or control sample after the incubation period at 44.5 ± 2 °C for 24–48 h was considered positive for *E. coli.*

Tryptone Bile X-Glucuronide (TBX) agar is a chromogenic selective medium used to isolate and identify *E. coli* containing bile salts that act as selective agents inhibiting most Gram-positive bacteria^43^. A 10 µL of positively confirmed tubes were cultured on TBX agar media (99.90%, ready-made media from Sigma-Aldrich) and incubated at 44 ± 1 °C for 18–24 h. After the incubation, the colour of the colonies was observed, with blue-green colonies indicating β-glucuronidase-positive *E. coli* and white colonies indicating β-glucuronidase-negative bacteria.

#### Measuring the release of Ag^+^ ions from BAg1 and BAg4 BG samples

BAg1 and BAg4 BG samples were selected to monitor Ag^+^ ion release. A 0.2 g of BAg1 and BAg4 BG samples were incubated in 10 mL of deionized water separately as a control and in 10 mL of SWW sample for 2, 4, 6, 12, and 24 h, one tube for each incubation time. Ag^+^ ion concentrations were measured (Atomic Absorption Spectrophotometer Buck Scientific Acusys 211 series, USA), by an air/acetylene flame system. The concentrations of Ag^+^ ions in the samples were determined in part per million (ppm). After that, the Ag^+^ ion release rate^44^ and percentages^45,46^ were calculated according to Eq. (3) and (4):

3 $$\text{Silver release \%}=\frac{\text{Amount released }(\text{ppm})}{\text{Total amount }(\text{ppm})}\text{x }100$$

4 $$\text{Silver release rate }(\text{ppm }/\text{h})=\frac{\text{Amount released }\left(\text{ppm}\right)}{\text{Time }\left(\text{h}\right)}$$

### Biological evaluation of the BAgX BG samples against *E. coli* O157:H7

#### Antibacterial activity assay

The agar well diffusion method was used to determine the antibacterial activity of the BG samples^47^. In brief, LB agar medium was prepared and inoculated with 50 µL of sterilized standard inoculum of *E. coli* O157:H7 (OD_600_ of ~ 0.7). After that, 1 cm-diameter wells were made in the LB agar medium using a sterile corkborer. After that, 50 mg of all types of BAgX BG powders were introduced separately into the wells under aseptic conditions either as silver-free BG as parent BG (BAg0) or as silver-doped BG (BAg1, BAg2, BAg3, and BAg4) samples. Then, 0.1 mL of sterile deionized H_2_O was added to the BG powders in each well. All the plates were preserved in the refrigerator at 4 °C for 1 h. Finally, the plates were incubated at 37 °C for 24 h, and the inhibition zones were measured. Moreover, amoxicillin (50 μg/mL) was used as a positive control for many bacteria. The experiments were carried out in triplicate, and the mean values were calculated. The growth percentage inhibition was calculated according to Eq. (5).

5 $$\text{Growth inhibtion \%}=\frac{\text{Inhibtion zone diamter of BAgX BG samples }(\text{mm})}{\text{Inhibtion zone diamter of amoxicillin }(\text{mm})}\text{x }100$$

##### Minimum inhibitory (MIC) and minimum bactericidal concentrations (MBC) of BAgX BG samples

The MIC is the lowest concentration of an antimicrobial agent that inhibits the visible growth of a microorganism after overnight incubation^48,49^. The MICs of the samples were commonly determined for each strain by the macro dilution broth method as described by the NCCLS^50^. The MICs of BAgX (X = 0, 1, 2, 3, and 4) BG samples were conventionally determined for *E. coli* O157:H7 by the serial dilution method of LB broth media. Since serial dilutions of all BAgX BG samples were prepared in bottles with various concentrations (50,000, 5000, 500, 50, and 5 ppm), the same inoculum volume (0.1 mL) of *E. coli* O157:H7 (OD_600_ of ~ 0.7) were added to all bottles. Then, the bottles were incubated at 37 °C, at 150 rpm, for 24 h. Each bottle was examined for growth and compared to the bacterial culture without BAgX treatments as a negative control. The growth of *E. coli* O157:H7 was determined by measuring the turbidity at OD_600_ with an ultraviolet–visible (UV–Vis) spectrophotometer (Jenway 7205, United Kingdom). The lowest concentration of BAgX required to inhibit *E. coli* O157:H7 (MIC) was recorded and plotted.

The MBC is the lowest concentration of antibiotic required to kill a particular bacterium^51^. The tubes were investigated to determine MIC values after 24 h of incubation. The MBC values were determined by sampling from all the macroscopically clear tubes after the first turbid tube in the series. Universally one dilution below the MIC was used for the levels to be assessed in the MBC assay^48^. A 50 µL from all cultures (used in the MIC test) was cultured onto plates of LB agar media. The plates were incubated for 24 h at 37 °C. The lowest concentration of BAgX BG required to kill *E. coli* O157:H7 (MBC) was recorded.

##### Detection of Ag-doped borate BG samples-resistant variants

The development of spontaneous resistance was conducted only for *E. coli* O157:H7 treated with BAg1, BAg2, BAg3, and BAg4 BG samples based on the MBC experiment^52^. Each 100 µL *E. coli* O157:H7 inoculum (OD _600_ of ~ 0.6–0.7) was inoculated into 10 mL LB broth medium. Treatments with variable concentrations (1X, 2X, and 3X MBC) of BAg1, BAg2, BAg3, and BAg4 BG were applied separately to every bacterial culture. The bottles were incubated at 37 °C, 150 rpm for 14 days incubation. Then, the optical density (OD_600nm_) of *E. coli* O157:H7 growth (CFU/mL) with BAg1, BAg2, BAg3, and BAg4 treatments in addition to the bacterial culture without BAgX treatments as a negative control in all bottles was recorded every two days by UV–Vis spectrophotometer. Parallel to spectrophotometry measurements, 50 µL treated cultures were cultured onto LB agar plates to detect the regrowth of *E. coli* O157:H7. Finally, the plates were incubated for 24 h at 37 °C, and the growth of *E. coli* O157:H7 was observed.

##### SEM analysis of *E. coli* O157:H7

To investigate the morphological cellular effect of the prepared BG samples against *E. coli* O157:H7 bacterial strain, SEM imaging (JEOL microscope JSM 6510 LV, Japan) was used. To obtain the SEM micrographs for both BAg0 and of BAg1, a 100 µL *E. coli* O157:H7 inoculum was added to every 10 mL LB broth medium provided with sub-MIC of BAg0 (5000 ppm) and of BAg1 (500 ppm) BG samples. The SEM micrograph *E. coli* bacterial strain (without treatment) was considered as a control. Then, the prepared bottles were incubated in a shaker at 150 rpm (37 °C) for 24 h. Thereafter, these bottles were centrifuged at 6000 rpm for 5 min and washed with a 1.0 M saline solution and this step was repeated three times. Finally, the supernatant was discarded, and the pellets were resuspended into the 4F1G Fixative (4% Formaldehyde and 1% Gluteraldehyde) for SEM imaging investigation.

### Statistical analysis

The data were initially evaluated for normality through the Shapiro–Wilk test and for homogeneity of variances via Levene’s test. Descriptive statistics, including the mean and standard deviation (mean ± SD), were computed to summarize the data. One-way ANOVA (SPSS Statistics program, V.27) was utilized for group comparisons in the in vitro antibacterial studies, followed by Tukey’s post hoc test to determine significant pairwise differences. Statistical significance was established at *p* < 0.05.

## Results and discussion

### Preparation of BAgX BG samples

**Fig. 2:**
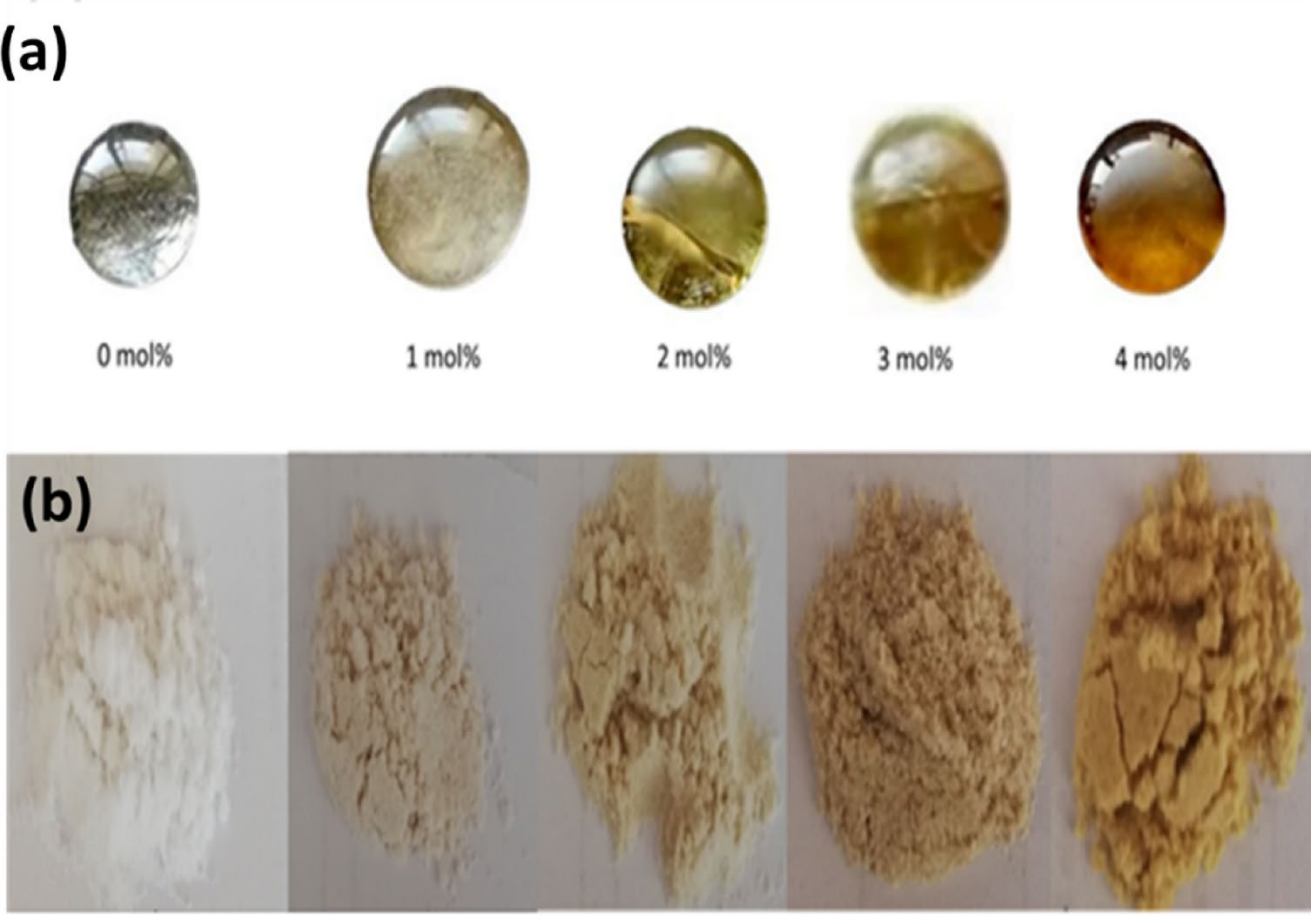
Digital photographs of BAgX bioglass samples doped with different concentrations of Ag_2_O (X = 0, 1, 2, 3, and 4 mol%) as (**a**) BAgX discs before grinding, and (**b**) the powder after grinding process.

Five types of silver-doped borate BG samples, BAg0, BAg1, BAg2, BAg3, and BAg4, were prepared by the melt-quenching method at high temperatures between 1100 and 1200 °C as spherical-shaped discs (Fig. 2). After the preparation, the grinding process would occur for all the BG samples to give powders an acceptable size. Finally, the colour intensity of the prepared samples elevated with increasing the concentration of Ag_2_O doped in the BG samples. Furthermore, during the grinding process, the hardness of the prepared samples increases with increasing the concentration of Ag_2_O in the BAgX BG samples investigated. It was noticed that BAg4 BG was the optimum hardness, followed by BAg3, BAg2, BAg1, and BAg0 BG samples.

### Characterization of the prepared BAgX BG samples

#### FT-IR spectroscopic analysis

**Fig. 3:**
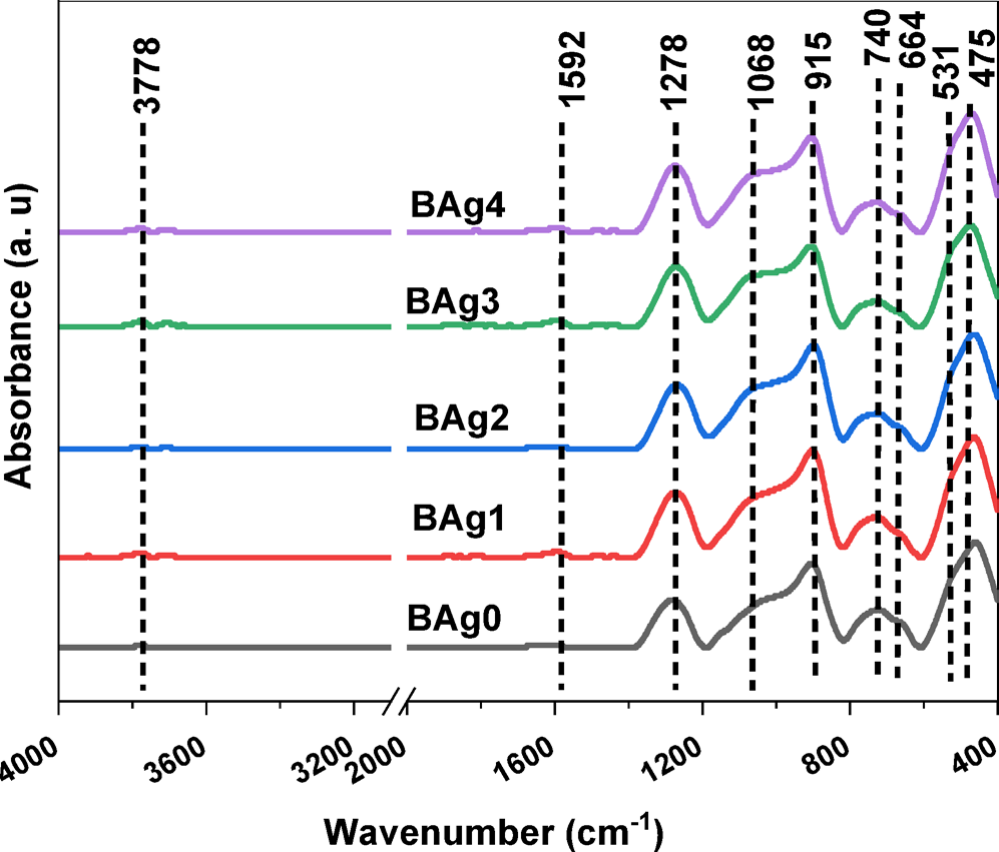
FTIR absorption spectra of BAg0 parent bioglass sample (free of Ag_2_O), BAg1, BAg2, BAg3, and BAg4 bioglass samples.

**Fig. 4:**
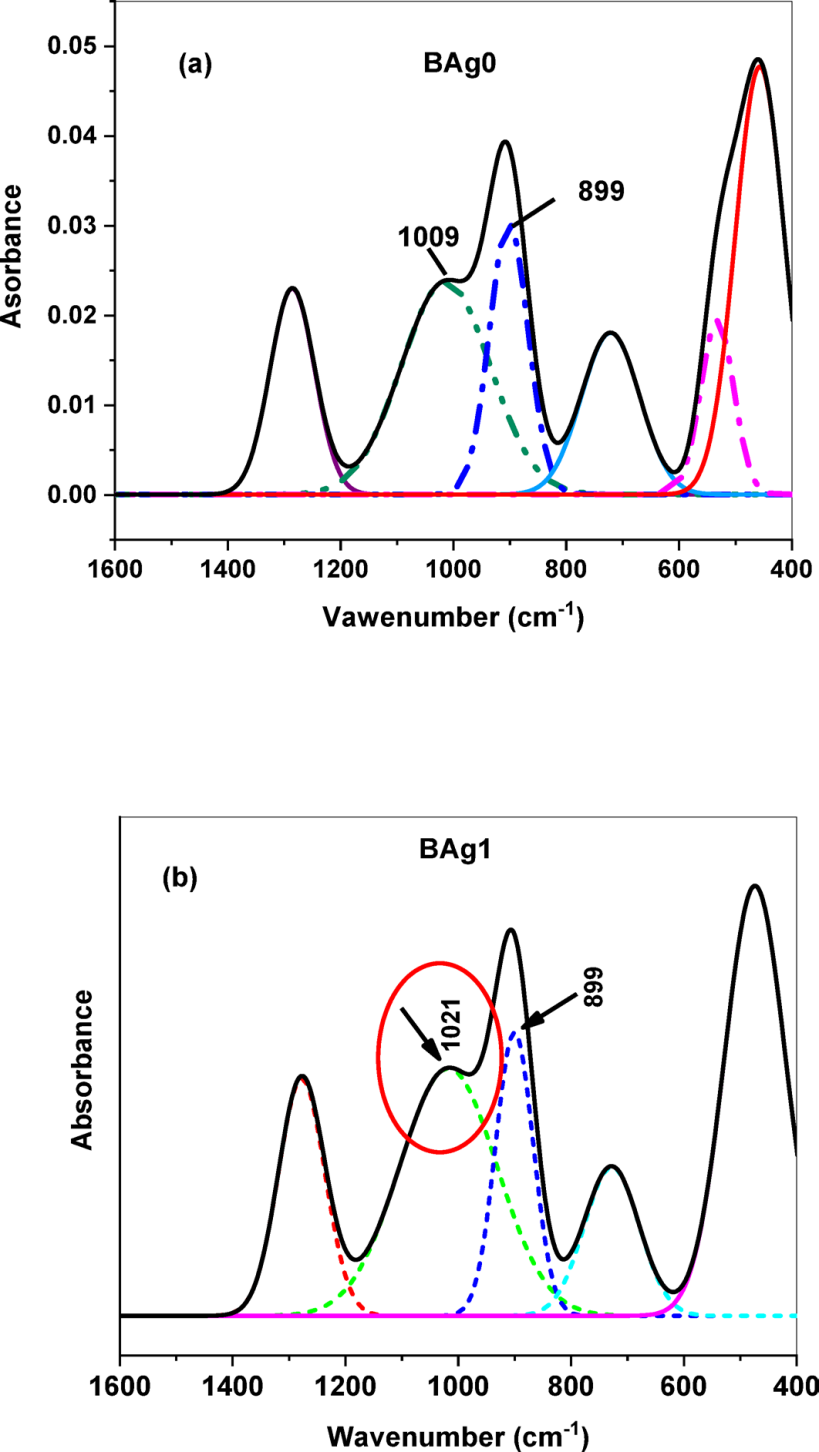
The deconvolution of the FTIR spectrum of the BAg0 parent bioglass sample (free of Ag_2_O) (**a**), and the BAg1 bioglass sample (**b**).

Figure 3 represents the FTIR absorption spectra of the examined BG samples measured between 400 and 4000 cm^-1^. The several types of functional groups present in the prepared BAgX BG samples were identified by FTIR analysis of the BG samples investigated. The base silver borate glass’s infrared spectrum exhibits the following distinctive characteristics: i) a narrow band with a peak at roughly 664 cm^-1^; ii) a small peak at roughly 475 cm^-1^; iii) a distinct medium band at roughly 740 cm^-1^; iv) a broad band extending from roughly 800 to 1200 cm^-1^ (with two peaks at about 901 and 1009 cm^-1^ when resolving the broadband peak broadband peak as represented in Fig. 4; vi) a tiny peak measuring 1592 cm^-1^, and finally vii) an additional small peak measuring 3775 cm^-1^. The assignment of the peak positions of the investigated BG is depicted in Table. 2.

**Table 2:** Assignment of the peak positions of the FTIR spectra of Ag_2_O-B_2_O_3_-Na_2_O-CaO-P_2_O_5_ BG samples.

Peak position [cm^-1^]	Assignment	Reference
400–500	Vibrations of metal cations Total symmetric bending vibrations of BiO_3_ units	^53^ ^56^
500–600	PO_4_^3−^ bending vibrational modes	^53,54,57^
740	Bending of the B-O-B linkage Oxygen bridges between one tetragonal and one trigonal boron atoms	^54,58^
850–900	Tri-, penta-, and diborate group (stretching) vibrations of BO4 group	^58,59^
1009	B-O-B vibration of BO_4_ group	
1020–1035	Tri-, penta-, tera-, and diborate group (stretching) vibrations of BO4 group	
1021	Ag–O–B bond connecting borate groups	
1200–1600	Stretching of various trigonal BO_3_ units	
1592	Crystal water with H–O-H bending mode	^56^
3700–3800	Free hydroxyl groups (–OH stretching)	^60^

The assigned band at 475 cm^-1^ may be attributed to the vibrations frequency of metal cations^53^. Furthermore, the structure of BG samples is affected in the same way by subsequent additions of Ag_2_O (0–4 wt%), as described by^33,54^. It can be noticed that the deconvolution of the band around 1000–1100 cm^-1^, the splitting of the peak into 899 and 1009 cm^-1^ for BAg0 sample. The chemical shift of the vibrational frequency from 1009 to 1021 cm^-1^ for BAg1 sample. respectively. This chemical shift suggests the interaction of silver ions (Ag⁺) with the phosphate groups (P₂O₅) in the borate glass matrix, therefore altering the local bonding environment and network connectivity^53^. Moreover, the most significant change in the peaks at 957–817 cm^−1^ was observed in the samples, indicating that an increase in the amount of Ag_2_O leads to the dominance of asymmetric BO_3_ (borate tetrahedral containing AgBO) in the glass network structure, as reported by Abdallah, Meikhail [55].

##### XRD analytical technique

**Fig. 5:**
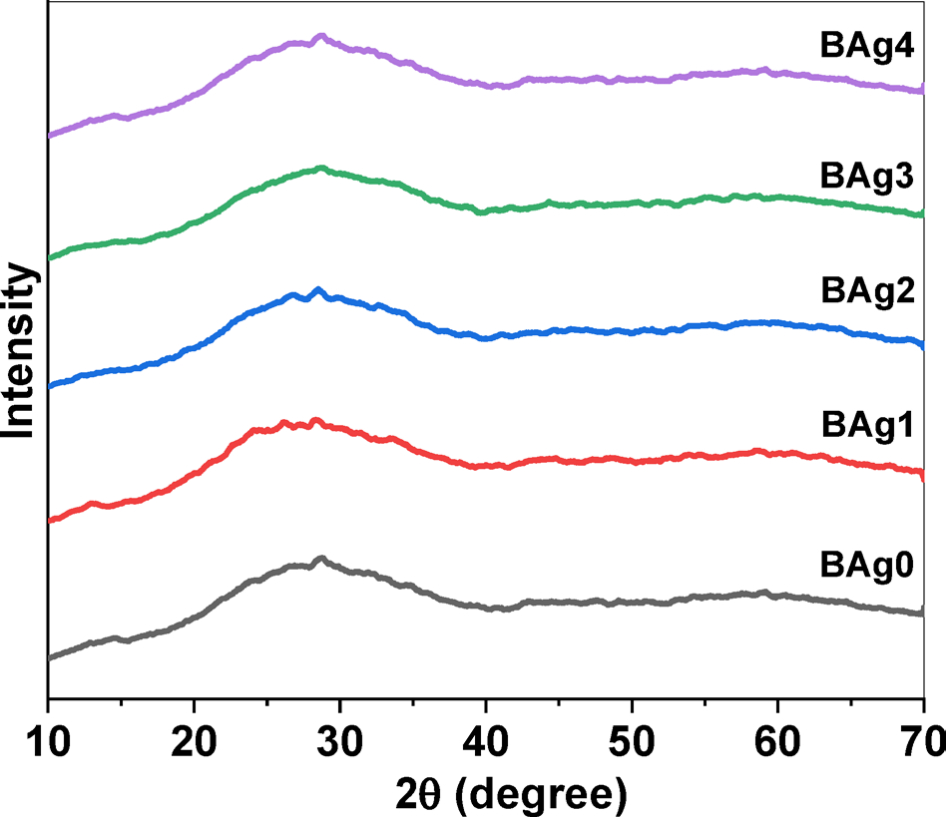
XRD spectra of BAg0 parent bioglass (free of Ag_2_O) and BAg1, BAg2, BAg3, and BAg4 bioglass samples.

XRD pattern analysis was used to determine the crystallinity of the prepared BAgX. As observed in the XRD, silver samples (1- 4 mol%) have no identifiable peaks and/or bands, indicating that the BG samples being investigated are amorphous, with no signs of crystallization or verification outcomes from silver addition as shown in Fig. 5. Even with increasing Ag_2_O content, the diffraction patterns showed that all the produced BG samples had an amorphous structure. A broad hump from 20 to 35° shows that the BG structure is amorphous rather than crystalline ‘

##### SEM imaging results for BAg0 and BAg1 BG samples

**Fig. 6:**
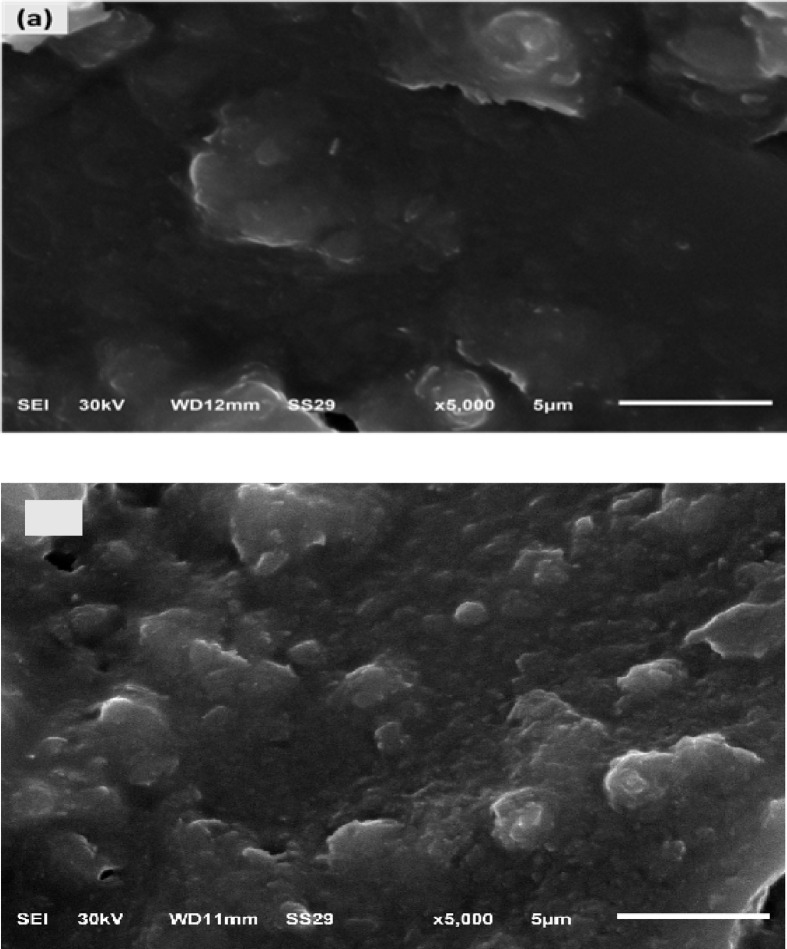
SEM micrographs of (**a**) parent BAg0 and (**b**) BAg1 bioglass samples (magnification× 5000 and scale bar of 5 µm).

The surface morphology is analyzed by SEM imaging for the prepared BG samples. The SEM image for the parent BG BAg0 sample is represented in Fig. 6a. The evidence of the accumulation of the silver nanoparticles (Fig. 6b) confirms the formation of the Ag nanostructure. These findings indicate that heat treatment helps modify the particles to the nanostructure dimension. This, in turn, will strongly affect the antibacterial measurements, as discussed later. The SEM images examine the surface smoothness, which is almost homogeneous and exhibits no evidence of crystallization. The BG system is amorphous, as no grain boundaries are discernible on the surface^61^.

##### EDX Spectroscopy of BAg1 BG sample

**Fig. 7:**
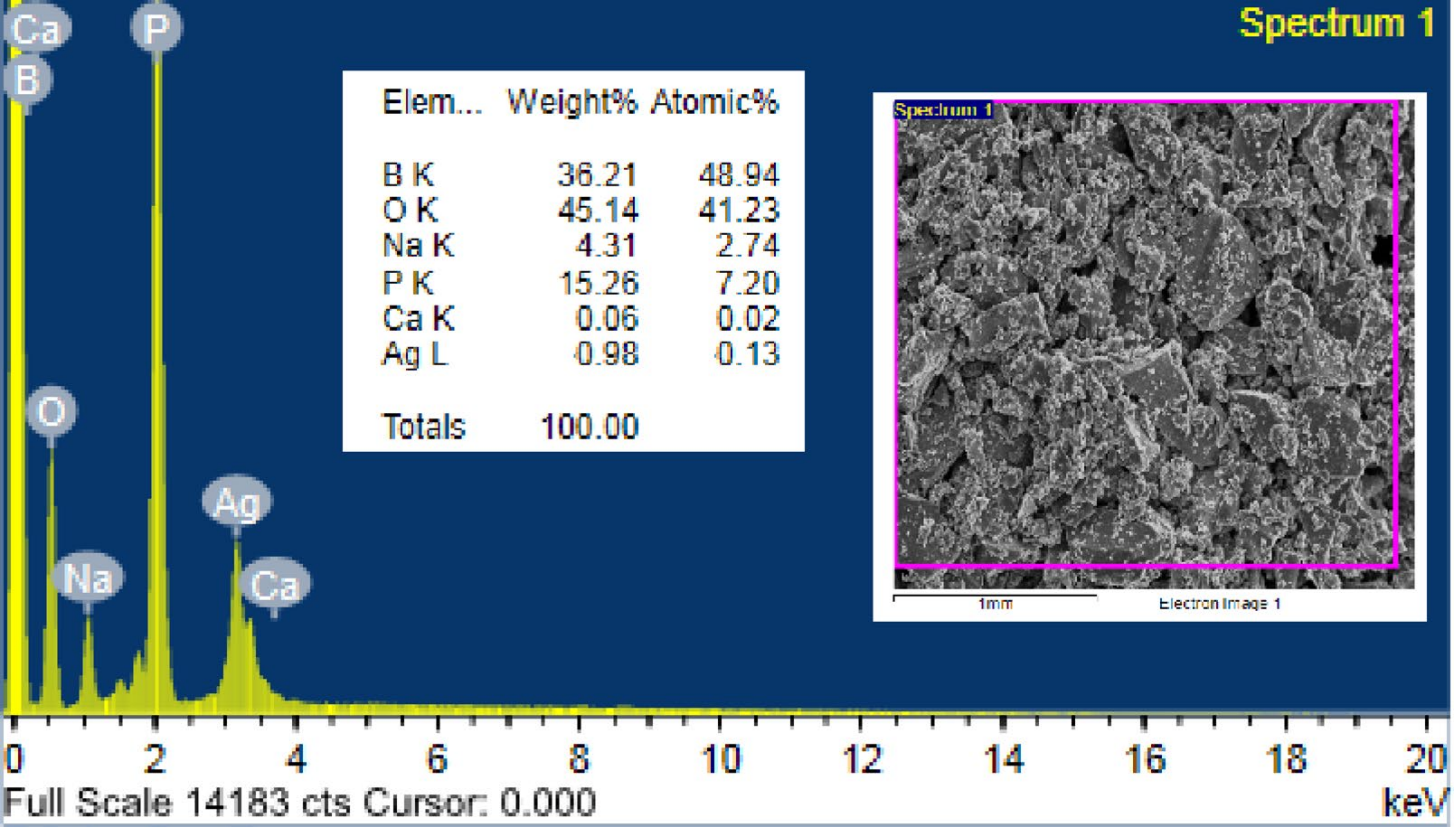
EDX spectrum for the BAg1 bioglass sample.

EDX is a powerful analytical technique that allows for the accurate elemental analysis and chemical characterization of the samples. It also reveals the ratio of different structure materials, whether natural or in oxide forms. Figure 7 shows the EDX results that justify the presence of specific peaks at energies 3.60, 0.31, 1.01, 1.92, 0.15, 0.54, and 3.1 keV related to Ca Kα, Ca Lα, Na Kα, P Kα, B Kα, O Kα, and Ag Lβ, respectively. Furthermore, based on the analysis of the sample’s elemental composition using the EDX technique, EDX spectra encompass a diverse array of chemical elements, including boron (B), oxygen (O), sodium (Na), calcium (Ca)^62^, phosphorus (P) and silver (Ag)^63^ The findings validate that the constituents present in the BG sample were congruent with those utilized in the BAgX BG samples throughout its synthesis^64,65^.

##### DLS study of BAg0 and BAg1 BG samples

**Fig. 8:**
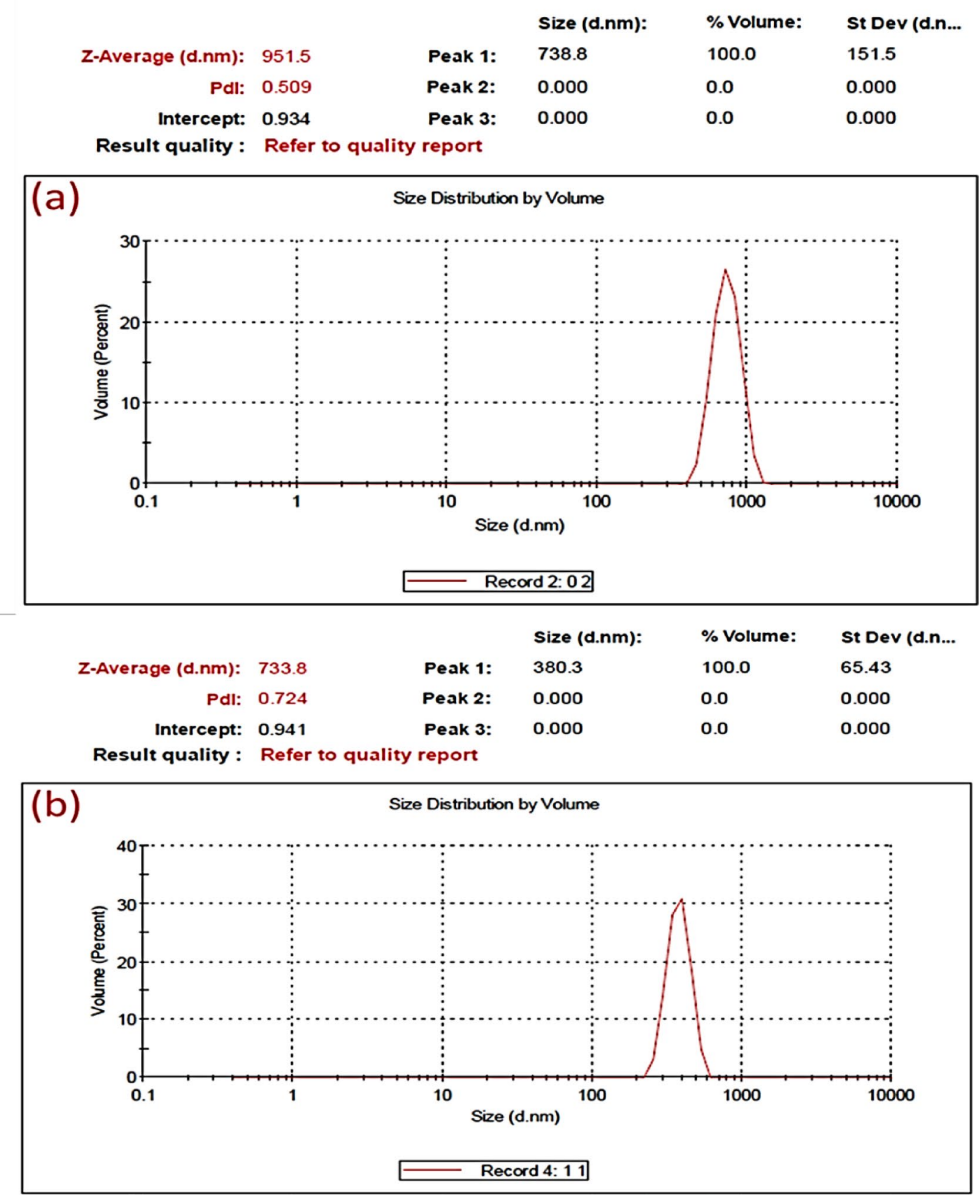
The DLS spectrum of parent BAg0 (borate bioglass free of Ag_2_O) bioglass sample (**a**) and BAg1 bioglass sample (**b**).

The average hydrodynamic particle size can be examined using the DLS technique. It can be noticed that the average particle size of the BAg0 parent BG sample was 738 ± 151 nm, (Fig. 8a). Controversy, the particle size distribution of the borate silver BG sample (BAg1) was 380 ± 65 nm as shown (Fig. 8b). This seems because the BG needs heat treatment to activate metallic particles. Hence, it is essential to ascertain the approximate temperature range for heat treatment. Therefore, the BG transition temperature is designated for amorphous substances. Where, above this temperature, the material attains a rubber-like state, allowing dopant ions to move and form nanocrystals^66^. Generally, small Ag NPs exhibited greater phytotoxicity than larger particles, as observed by Korani et al. (2015)^67^. Also, Dang et al. (2020) demonstrated how plants absorb Ag NPs by assessing the relative bioavailability of Ag NPs compared to Ag^+^^68^.

### Effect of the BAgX BG samples on the growth of coliform bacteria in SWW samples

#### MacConkey agar plates

**Fig. 9:**
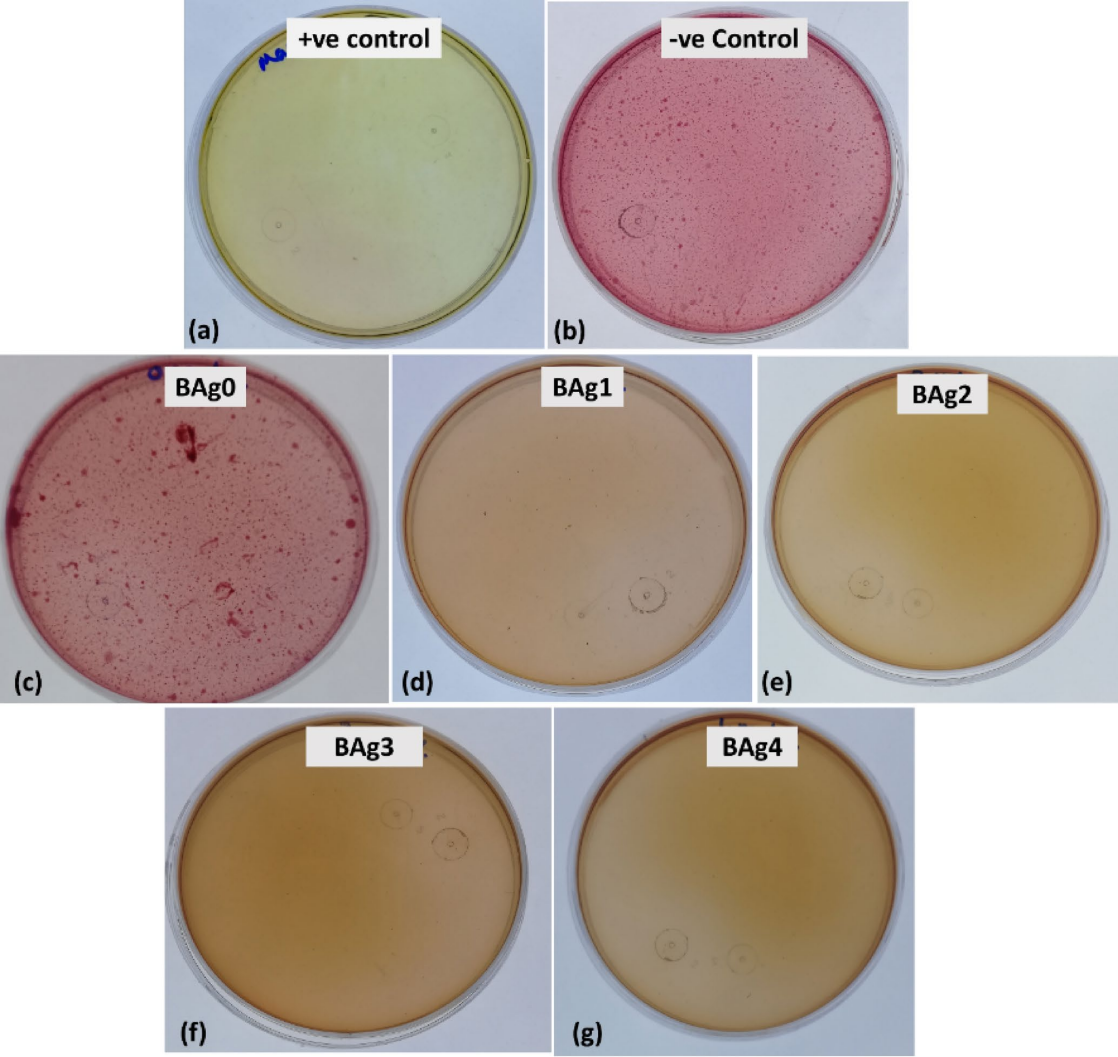
The photos of the coliform bacteria detection in SWW samples as pink colonies on MacConkey agar plates after treatments with BAgX bioglass samples compared to positive and negative controls. (**a**) positive control (sample treated with (Ca (ClO)_2_), (**b**) negative control (untreated sample), (**c**) BAg0 parent bioglass, (**d**) BAg1, (**e**) BAg2, (**f**) BAg3, and (g) BAg4 treatments.

The BAgX BG samples were applied for the treatment of the coliform bacteria in SWW samples compared to SWW samples treated with Ca (ClO)_2_ as a positive control in addition to the untreated sample as a negative control. Figure 9 represents the photos of the coliform bacteria detection in SWW samples as pink colonies on MacConkey agar plates after treatments with BAgX BG samples compared to positive and negative controls. Generally, compared to both controls, all samples treated with BAg1, BAg2, BAg3, and BAg4 BG samples (Fig. 9d–g) exposed complete inhibition of the coliform bacteria, so all agar plates showed no bacterial colonies recording 100% removal, the same action recorded in a SWW sample treated with Ca (ClO)_2_. On the other hand, the BAg0 parent BG sample (Fig. 9) showed the lowest removal percentage of a value of 20.94% (Table 3).

**Table 3:** Bacterial** c**ell colony forming unit count (CFU/mL) and removal percentages of coliform bacteria from SWW samples treated with BG samples compared to positive and negative control samples.

Treatments		CFU/ mL	Removal (%)
Control	Negative	148 × 10^2^	0.00
	Positive Ca (ClO)_2_	0.00	100.00
BAgX	BAg0	117 × 10^2^	20.94
	BAg1	0.00	100.00
	BAg2	0.00	100.00
	BAg3	0.00	100.00
	BAg4	0.00	100.00

The BAgX BG samples at a 20 mg/mL concentration were applied to treat the coliform bacterial community in SWW samples. The antibacterial properties of BG have generated a lot of attention in biological applications^69^. Bioactive glass had antibacterial efficacy against pathogenic *E. coli* at concentrations of 0.02–20 mg/mL, according to Balamurugan, Balossier [70]. The clustering of Ag^+^ ions caused by high Ag_2_O concentrations doped in bioactive glass was the reason for the decreased antibacterial activity of Ag_2_O-doped BG as reported by Kunkalekar, Prabhu [71].

In both basic and applied research in aquatic microbial ecology, as well as in the development of parameter-based technologies for the assessment of drinking water and domestic wastewater quality for reuse, the enumeration technique of fecal bacteria is essential^72^. The World Health Organization’s (WHO) standards specifications (IS: 10500-2012) state that the total coliform count method has been widely utilized as a water safety indicator^73^. Drinking water should have a coliform and *E. coli* limit of 0/100 mL, whereas domestic and recreational water should have a limit of 126 CFU/100 mL^74,75^. Coliform counts on MacConkey agar plates, a water quality indicator that serves as a crucial indicator of the pathogens that most frequently cause diarrhoea, typhoid, and a variety of other enteric diseases, are used to track the strength of the fecal matter in SWW^76^.

MacConkey agar is a selective medium that separates Gram-positive bacteria from Gram-negative bacteria because it contains bile salts that interact with the lipid components of the Gram-positive bacterial cell membrane, disrupting it and causing cellular contents to leak out. This denatures the bacterial proteins and interferes with the bacteria’s ability to function and with several metabolic processes, including DNA replication, protein synthesis, and energy production, which ultimately results in cell death^77^. However, the cell wall structure of Gram-negative bacteria, which consists of periplasm, a thin peptidoglycan layer, and inner and outer membranes, allows them to grow on MacConkey agar medium^78^. MacConkey agar can be used for both therapeutic and research purposes because of its unique and selective qualities^79^. Lactic acid, the primary organic acid produced during lactose fermentation, lowers the pH of the agar. Phenol red, a pH indicator found in MacConkey agar media, turns pink in acidic environments. Hence, lactose-fermenting Gram-negative bacteria will produce pink colonies, whereas non-lactose fermenters will produce opaque, off-white colonies^80^.

#### Inside fermentation tubes

##### Presumptive test

Generally, the number of positive tubes increases with the increase of Ag_2_O concentrations doped in borate-based BG samples. Compared to untreated samples or control after 24 and 48 h (Fig. S2 a and 3a), all samples of SWW treated with BAg1, BAg2, BAg3, and BAg4 accepted for application or reuse where the results of MPN/100mL were less than 5000 value. On the contrary, samples treated with BAg0 parent BG, free of Ag_2_O, were not accepted for reuse (Table. 4). In the case of the BAg0 parent BG sample, after 24 h treatment with SWW, all tubes of all groups inoculated with 5, 1, and 0.1 mL SWW turned yellow and produced gas (5.5.5), indicating the presence of coliform bacteria. Moreover, after 48 h from SWW treatment, the intensity of the colour would increase altogether in all tubes (5.5.5) of all groups (Figs. S2b and 3b). The BAg0 parent BG sample detected the most probable number, reaching > 160,000 MPN/100 mL after 24 and 48 h treatments.

**Table 4:** MPN index of coliform bacterial growth in SWW samples using MacConkey broth purple media after 24 and 48 h treatments by BAg0 parent BG sample, borate BG free from Ag_2_O, and borate BG doped with different concentrations of Ag_2_O, BAg1, BAg2, BAg3, and BAg4 BG samples.

Sample volume	1^st^ group 5 mL					2^nd^ group 1 mL					3^rd^ group 0.1 mL					Combination of positives of coliform bacteria	MPN /100 mL	Acceptance
Tubes	**1**	**2**	**3**	**4**	**5**	**1**	**2**	**3**	**4**	**5**	**1**	**2**	**3**	**4**	**5**			
After 24 h																		
Control	**+**	**+**	**+**	**+**	**+**	**+**	**+**	**+**	**+**	**+**	**+**	**+**	**+**	**+**	**+**	5.5.5	˃ 160,000	No
BAg0	**+**	**+**	**+**	**+**	**+**	**+**	**+**	**+**	**+**	**+**	**+**	**+**	**+**	**+**	**+**	5.5.5	˃ 160,000	No
BAg1	**−**	**−**	**−**	**−**	**−**	**−**	**−**	**−**	**−**	**−**	**−**	**−**	**−**	**−**	**−**	0.0.0	0.00	Yes
BAg2	**−**	**−**	**−**	**+**	**−**	**−**	**−**	**−**	**−**	**+**	**−**	**−**	**−**	**−**	**−**	1.1.0	400	Yes
BAg3	**−**	**−**	**−**	**−**	**−**	**+**	**−**	**−**	**−**	**−**	**+**	**−**	**−**	**−**	**−**	0.1.1	360	Yes
BAg4	**−**	**−**	**−**	**+**	**−**	**−**	**−**	**−**	**−**	**−**	**+**	**−**	**−**	**−**	**−**	1.0.1	400	Yes
After 48 h																		
Control	**+**	**+**	**+**	**+**	**+**	**+**	**+**	**+**	**+**	**+**	**+**	**+**	**+**	**+**	**+**	5.5.5	˃ 160,000	No
BAg0	**+**	**+**	**+**	**+**	**+**	**+**	**+**	**+**	**+**	**+**	**+**	**+**	**+**	**+**	**+**	5.5.5	˃ 160,000	No
BAg1	**−**	**−**	**−**	**−**	**−**	**−**	**−**	**−**	**−**	**−**	**+**	**−**	**−**	**−**	**−**	0.0.1	180	Yes
BAg2	**+**	**+**	**−**	**−**	**−**	**−**	**−**	**−**	**+**	**−**	**−**	**−**	**−**	**−**	**−**	2.1.0	680	Yes
BAg3	**−**	**+**	**+**	**−**	**−**	**+**	**−**	**−**	**−**	**−**	**+**	**−**	**−**	**−**	**−**	2.1.1	920	Yes
BAg4	**−**	**+**	**−**	**−**	**+**	**+**	**+**	**−**	**−**	**−**	**−**	**−**	**−**	**+**	**+**	2.2.2	1400	Yes

MPN/100mL ≤ 5,000 MPN is accepted, MPN/100mL > 5,000 MPN is not accepted.

In the case of the BAg1 BG sample, after 24 h treatment with SWW, no change in colour (i.e., no yellow colour) and also no gas formation would occur in all tubes of all groups that were inoculated with 5, 1, and 0.1 mL of SWW (0.0.0) recording 0 MPN/100 mL. After 48 h, only one tube in the third group (0.0.1) that was inoculated with 0.1 mL of SWW sample treated with BAg1 changed to a yellow colour and produced gas in Durham’s tube recording 180 MPN/100 mL (Figs. S2c and 3c).

In the case of the BAg2 BG sample, after 24 h treatment with SWW, yellow colour and gas formation in Durham’s tubes would occur only in two tubes (one in the first group and one in the second group, 1.1.0), recording 400 MPN/100 mL. After 48 h, the number of positive tubes increases from 2 to 3 tubes (two in the first group and only one in the second group, 2.1.0), recording 680 MPN/100 mL (Figs. S 2d and 3d).

In the case of the BAg3 BG sample, after 24 h treatment with SWW, yellow colour and gas formation in Durham’s tubes would occur in only two tubes (one in the second group and one in the third group, 0.1.1), recording 360 MPN/100 mL. After 48 h, the number of positive tubes increased from 2 to 4 tubes (two in the first group, only one tube in the second group, and one tube in the third group, 2.1.1), recording 920 MPN/100 mL (Figs. S 2e and 3e). In the case of the BAg4 BG sample, after 24 h treatment with SWW, yellow colour and gas formation in Durham’s tubes would occur in only two tubes (one in the first group and one in the third group, 1.0.1), recording 400 MPN/100 mL. After 48 h, positive tubes increased from 2 to 6 (two tubes in the first group, two in the second group, and two in the third group, 2.2.2), recording 1400 MPN/100 mL (Fig. S 2f. and 3f).

Results revealed that the best molar ratio of Ag_2_O was 1 mol%. That indicated the increase of Ag_2_O concentration in the doped BG samples; the number of positive presumptive test tubes increased after the 48 h incubation period, i.e., a low rate of growth. The dual role of Ag_2_O as a biocidal metallic ion and B_2_O_3_ implemented in the BAg1, BAg2, BAg3, and BAg4 BG samples would be helpful in making the glass wider spectrum in their antibacterial activity. Liu, X., et al., (2010) confirmed that borate (B_2_O_3_) alone in BG inhibited the growth of *E. coli*, *S. aureus, Shigella sonnei*, *Staphylococcus epidermidis, Vibrio natriegens* and *Serratia marcescens*^81^. Additionally, doping Ag_2_O at crucial quantities, 0.2 -1 mol%, improved the borate-based BG system’s antibacterial activity^64^. In order to analyze the quality of SWW for reclamation and reuse, the bacteriological analysis was conducted by determining the MPN and then assessing the concentration of coliform bacteria in a sample. There were two stages to the MPN technique which are presumptive and confirmed tests^82^.

Bromocresol purple indicator, which turns from purple to yellow when coliform bacteria are presumed to be present in MacConkey broth, offers a more accurate and sensitive sign of acid generation^83^. In the MacConkey broth medium, distinct bacterial species can be distinguished by their varying rates of growth, even within lactose fermenters. Because of this, MacConkey broth is an effective method for identifying and isolating bacterial species from SWW^84^. After treatment, the suitability of SWW is evaluated with respect to permissible coliform bacterial concentrations or MPN (Table. 4), which must be restricted to the ultimate destination. These are determined by rules, regulations, or the highest amount that is allowed^85^. The fecal coliform count in raw sewage is generally between 10^6^ and 10^8^ MPN/100 mL, according to Seth [86]. Coliform bacteria do not cause disease; rather, they are a sign that SWW could contain pathogenic organisms of fecal origin. For a water treatment plant in India, the Central Pollution Control Board recommended that the total coliform standard at the intake point be set at 5000 MPN/100 mL^86–88^.

#### Confirmation test for *E. coli* growth in EC broth and on TBX agar media

##### In EC broth tubes

**Fig. 10:**
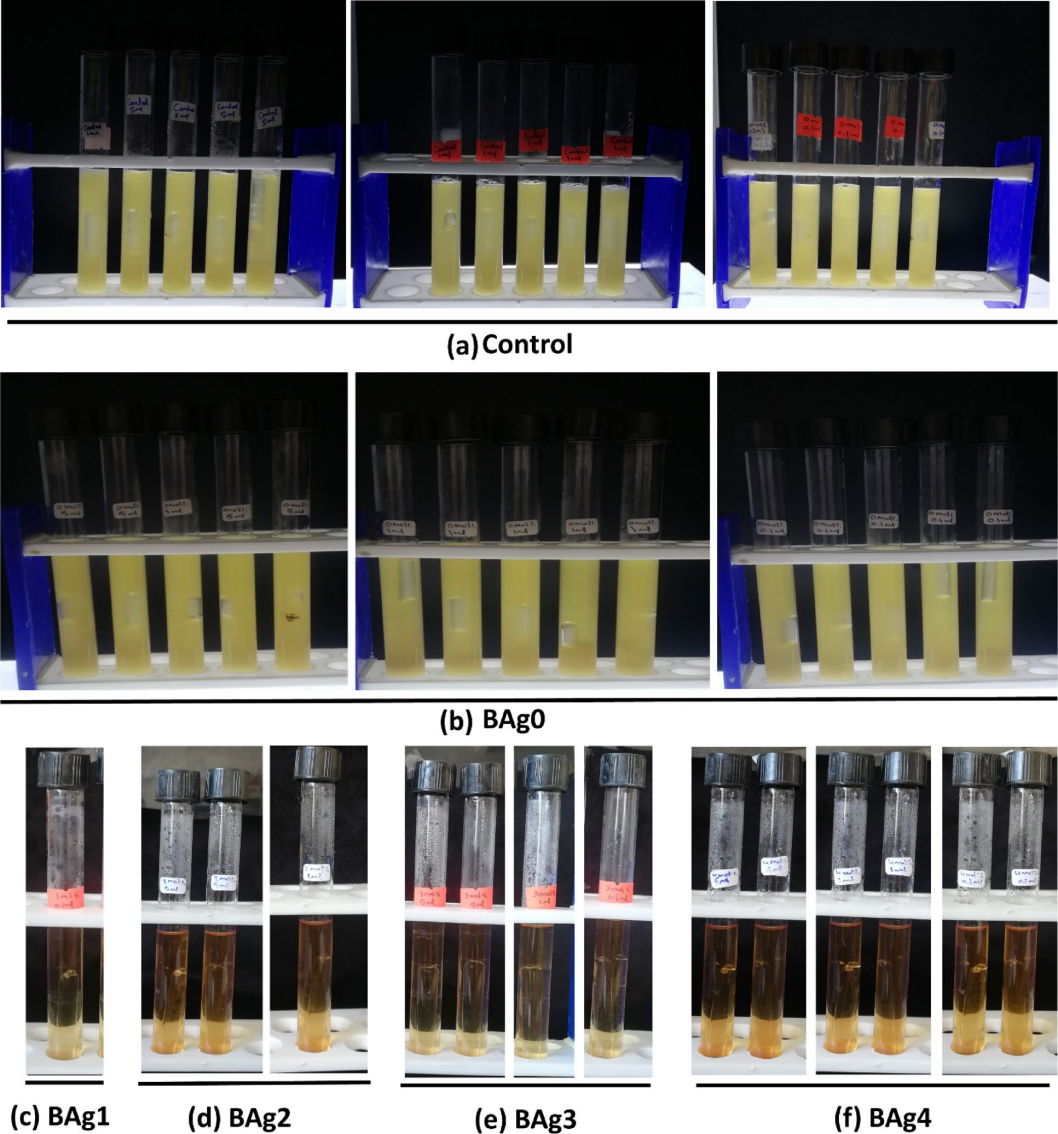
Digital photos of the confirmation test tubes for the presence of *E. coli*, as a fecal indicator, in EC broth tubes that were inoculated from positively presumptive test tubes of (**a**) untreated or control sample, (**b**) BAg0, (**c**) BAg1, (**d**) BAg2, (**e**) BAg3, and (**f**) BAg4 treatments.

The presence of *E. coli* in the coliform bacterial mixture of SWW was confirmed by subculturing positively presumptive test tubes in EC broth media as selective media for *E. coli* (Fig. 10a). Compared to untreated or control samples, the antibacterial activity of BAgX BG samples against *E. coli* was significantly improved by adding Ag_2_O content compared to BAg0 parent BG, free of Ag_2_O (Fig. 10b). The presence of *E. coli* is confirmed by the production of gas and the turbidity of the yellow colour of EC broth media. The absence of *E. coli* growth in EC broth media, with no lactose fermentation, would be caused by the antibacterial action of BAg1, BAg2, BAg3, and BAg4 BG samples (Fig. 10c–f). compared to the BAg0 parent BG sample, which has no antibacterial action against the fecal indicator *E. coli* that was included in a coliform bacterial mixture of SWW samples. Table 5 depicts the presence or absence of *E. coli* in the confirmed tubes that inoculated from positively presumptive test tubes of untreated (control sample) and treated BAg0, BAg1, BAg2, BAg3, and BAg4 BG samples.

**Table 5:** Presence or absence of *E. coli* in the confirmed tubes that inoculated from positively presumptive test tubes of untreated or control samples and samples treated with parent BAg0, BAg1, BAg2, BAg3, and BAg4 BG samples.

Treatments	No. of positive tubes in 1^st^, 2^nd^ and, 3^rd^ groups		Presence of *E. coli*
	Presumptive test	Confirmation test	
Control (untreated)	5.5.5	5.5.5	**+**Ve
BAg0	5.5.5	5.5.5	**+**Ve
BAg1	0.0.1	**−**.**−**.0	**−**Ve
BAg2	2.1.0	0.0.**−**	**−**Ve
BAg3	2.1.1	0.0.0	**−**Ve
BAg4	2.2.2	0.0.0	**−**Ve

(**−**) Sign means tubes didn’t expose to the confirmation test because they showed negative results in the presumptive test.

TBX agar plate

**Fig. 11:**
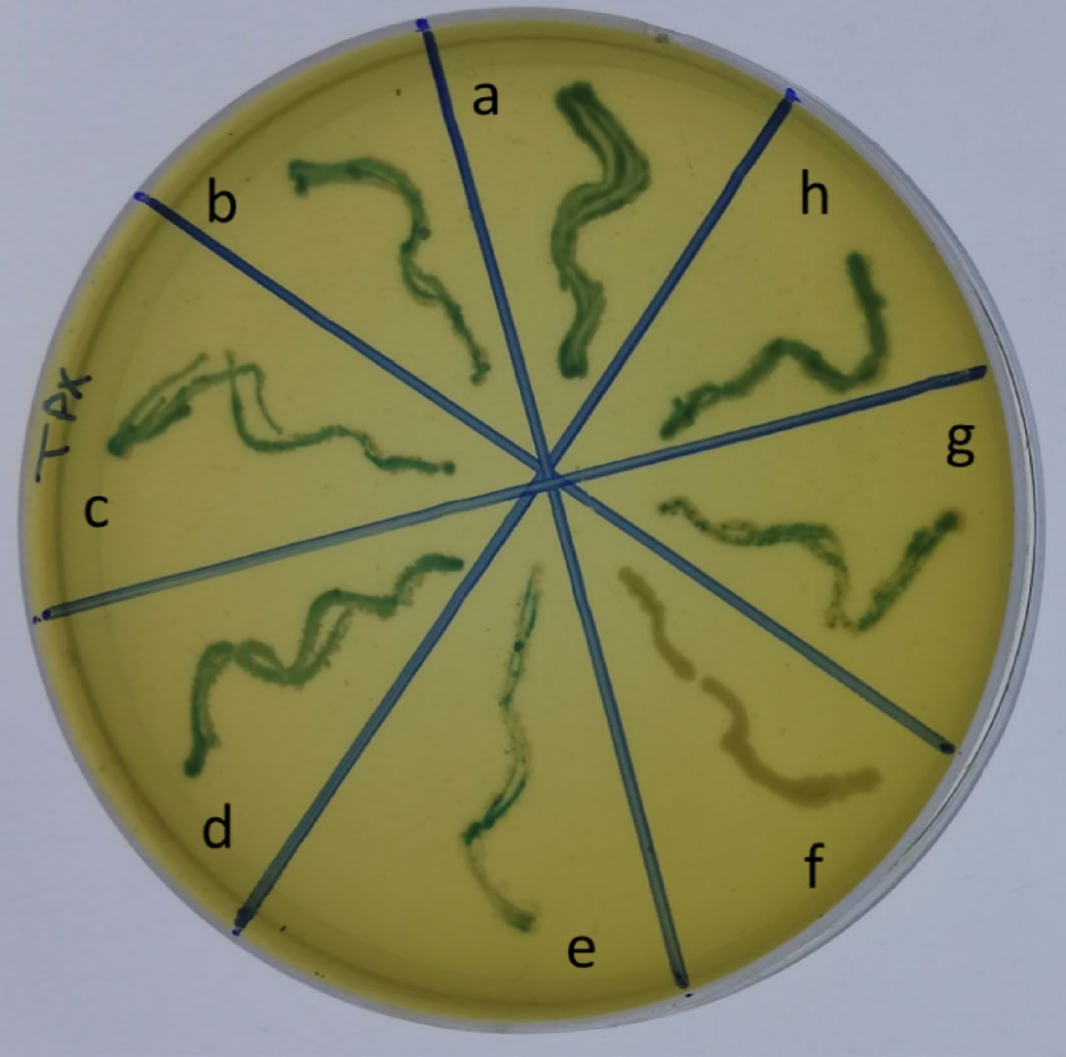
The distribution of *E. coli* on the TBX aga plate appeared as blue-green colour compared to the other fecal thermotolerant coliform bacteria appearing as white colour.

The positively confirmed EC broth tubes, containing SWW samples treated with BAg0 parent BG, were sub-cultured on the TBX agar plate as also a selective medium. After subculturing some positively confirmed tubes (8 tubes), the distribution of *E. coli* showed variable patterns on the TBX agar plate. The distribution of *E. coli* was dominant in one tube (Fig. 11a) with a dominant blue-green colour, intermediate in four tubes (Fig. 11b–d,h), and low frequency in two tubes (Fig. 11e,g). In contrast, the distribution of *E. coli* was rare in only one tube (Fig. 11f) with a dominant white colour.

Generally, the blue-green colour indicates the presence of *E. coli* that have β-glucuronidase that degrades β-glucuronide in TBX agar media, forming a blue-green colour on the plate. On the other hand, the white colour indicates the presence of other fecal thermotolerant coliform bacteria in the wastewater sample. Estimating the microbiological quality of surface water and the risk of human illness following exposure to it depends heavily on the presence of *E. coli* bacteria^43^. Gram-negative coliform bacteria were separated from *E. coli* that ferment lactose at 44.5 °C as thermotolerant fecal coliform bacteria in a medium containing bile salts using EC broth media to verify the existence of *E. coli* as fecal indicator bacteria^42^.

The highly antibacterial activity of the BAg1, BAg2, BAg3, and BAg4 BG samples against *E. coli* was recorded compared to the BAg0 parent BG sample, which has no antibacterial activity against the fecal indicator *E. coli* that was implemented in a coliform bacterial mixture of SWW sample*.* This is because of the highly antibacterial properties of both Ag_2_O and B_2_O_3_ implemented in BAg1, BAg2, BAg3, and BAg4 BG samples.

All *E. coli*, both Shiga-toxin (STEC) and non-STEC can be isolated and identified using TBX agar, a chromogenic selective medium that contains bile salts that function as selective agents that inhibit the majority of Gram-positive bacteria^89^. The presence of β-glucuronidase activity is indicated by the blue-green colour, while bacteria that are β-glucuronidase-negative are indicated by white colonies. Among bacteria, whole genome sequences are available for several Gram-positive bacteria, including *Lactobacillus gasseri*, *Staphylococcus* sp., *Clostridium perfringens*, *Staphylococcus aureus*, and *Thermotoga maritime*, and only one Gram-negative bacterium, *E. coli*, has genes encoding β-glucuronidase described^90,91^.

Compared to other coliform bacteria, *E. coli* was more prevalent in the wastewater that was released^43^. According to performance metrics, colony counts, and real-world applications, TBX seemed to be the best culture medium for counting *E. coli* in bathing water and other environments with high background bacterial concentrations, such as wastewater discharge. White other coliform colonies and blue-green *E. coli* colonies were enumerated on TBX from wastewater discharged after being incubated for 18–24 h at 44 °C. *Klebsiella oxytoca* and/or *K. pneumoniae* are two other thermotolerant coliform bacteria that can digest lactose at 44.5 °C^23,92^.

### The release of Ag^+^ ions from BAg1 and BAg4 BG samples

**Fig. 12:**
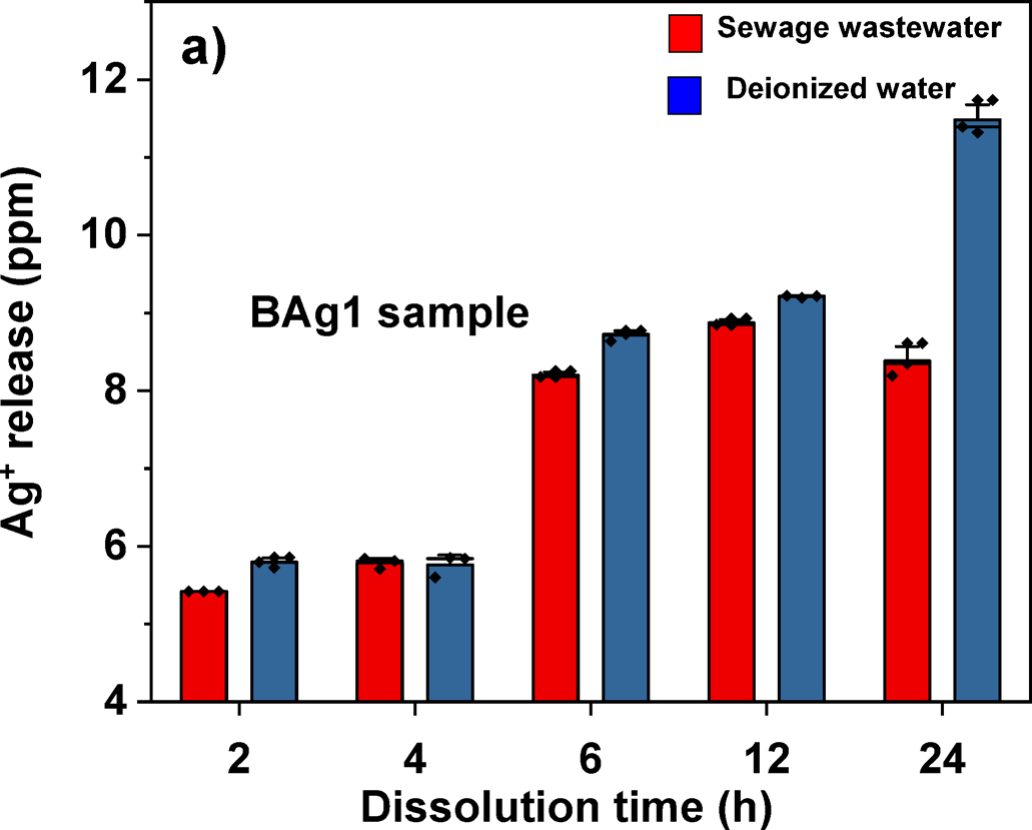

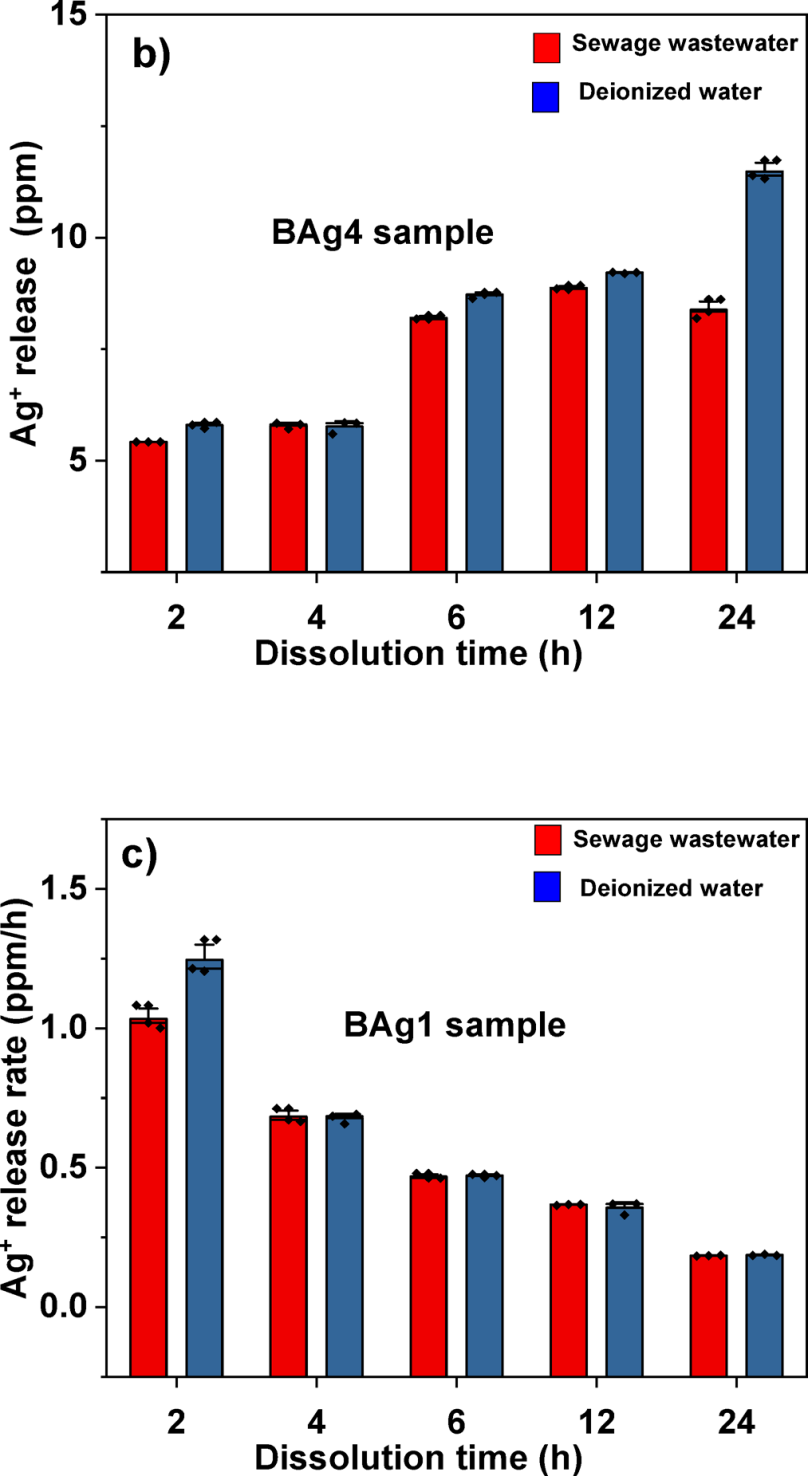

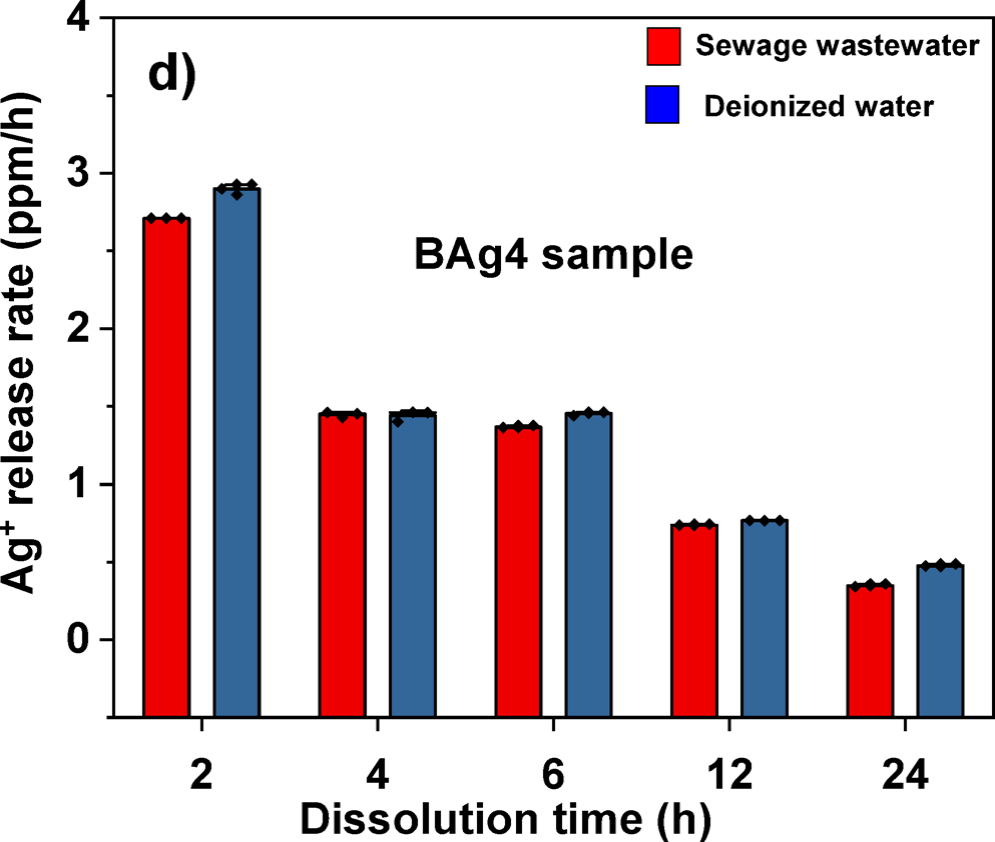
Ag⁺ release in SWW compared to deionized water from 2 to 24 h incubation time at 150 rpm and 37 ℃ from the BAG1 sample (**a**); (**b**) the BAG4 sample; (**c**) Ag⁺ release rate in SWW compared to deionized water from 2 to 24 h incubation time at 150 rpm and 37 ℃ for the BAG1 sample; and (**d**) the BAG4 sample.

The release and release rate of Ag⁺ ion from BAg1 and BAg4 samples was thoroughly evaluated over a 24-h period in both wastewater and deionized water, as depicted in Table. 6. Further, the release of Ag^+^ ions from the BAg1 BG sample, which is doped with the lowest concentration of Ag_2_O (1 mol%), as illustrated in Fig. 12a, was compared to that from the BAg4 BG sample, doped with the highest concentration of Ag_2_O (4 mol%), at a concentration of 20 mg/mL (Fig. 12b).

**Table 6:** The concentration (ppm) and the release percentage of Ag^+^ ions from BAg1 and BAg4 BG samples in SWW samples compared to deionized water from 2 to 24 h incubation period.

Sample	Time	The concentration of silver (ppm)		The release of Ag^+^ (%)		Ag^+^ release rate (ppm h^-1^)	
		BAg1	BAg4	BAg1	BAg4	BAg1	BAg4
Sewage wastewater	2h	2.07 ± 0.08	5.31 ± 0.10	1.04	0.66	1.035	2.655
	4h	2.73 ± 0.1	5.78 ± 0.07	1.37	0.72	0.683	1.445
	6h	2.81 ± 0.06	8.20 ± 0.05	1.41	1.03	0.468	1.367
	12h	4.41 ± 0.02	8.87 ± 0.05	2.21	1.11	0.368	0.739
	24h	4.43 ± 0.02	8.38 ± 0.21	2.22	1.05	0.185	0.349
Deionized water	2h	2.49 ± 0.13	5.79 ± 0.07	1.25	0.72	1.245	2.895
	4h	2.71 ± 0.07	5.76 ± 0.14	1.36	0.72	0.678	1.440
	6h	2.83 ± 0.03	8.71 ± 0.07	1.42	1.09	0.472	1.452
	12h	4.28 ± 0.27	9.21 ± 0.01	2.14	1.15	0.357	0.768
	24h	4.49 ± 0.06	9.55 ± 0.22	2.25	1.19	0.187	0.398

Each value is Mean ± SD (n = 3).

It can be noticed that the Ag concentration in BAg4 within SWW was consistently elevated compared to BAg1 across all measured time points, starting at 5.31 ± 0.10 ppm at the 2-h interval and reaching a maximum of 8.87 ± 0.05 ppm at the 12-h interval. Subsequently, it experienced a slight reduction to 8.38 ± 0.21 ppm at the 24-h interval. In contrast, BAg1 exhibited a linear increase from 2.07 ± 0.08 ppm at the 2-h to 4.43 ± 0.02 ppm at the 24-h.

To assess the release rate of Ag^+^ ions, the amount of Ag^+^ ions released per hour (ppm hr⁻^1^) was determined for both BAg1 and BAg4 in SWW, in comparison to deionized water, as shown in Fig. 12c,d. Initially, a greater amount was observed in BAg1 compared to BAg4; however, both samples exhibited a decrease over time. The concentration of Ag⁺ reached its highest during the initial stages (2 h) for both samples. BAg4 exhibited a more rapid release of silver ions at a rate of 2.655 ppm hr⁻^1^, whereas BAg1 released at a slower rate of 1.035 ppm hr⁻^1^, subsequently followed by a gradual decline over time.

Similar alterations were noticed in deionized water; BAg4 exhibited elevated Ag concentrations, reaching 9.55 ± 0.22 ppm at the 24-h, while BAg1 showed 4.49 ± 0.06 ppm. Initially, the release rates were high, as demonstrated in Fig. 12c,d, with values of 2.895 ppm hr⁻^1^ for BAg4 and 1.245 ppm hr⁻^1^ for BAg1 at the 2-h, respectively. The release percentages and rates exhibited similar trends, progressively decreasing over the subsequent 24-h period is shown in Table. 6. BAg1 consistently exhibits greater Ag⁺ release percentages than BAg4 at every time interval. The release percentage for both BG samples is somewhat greater in deionized water than in sewage wastewater, particularly at later intervals (e.g., 2.25% for BAg1 in deionized water at 24 h against 2.22% in sewage).

In addition, the release rate of Ag^+^ ions decreased with increasing leaching duration in SWW samples could be due to the reaction between Ag^+^ and Cl^−^, forming AgCl and affecting their dissolution^93^.

To elucidate the cytotoxicity of the BG samples, the average particle size of the BAg1 sample is 380 ± 65 nm, in contrast to the free silver BG sample (BAg0), which measures 738 ± 151 nm (Fig. 8), correlating with the cytotoxicity. It is known that the level of cytotoxicity caused by Ag NPs is influenced by their size, with nanoparticles smaller than 10 nm typically exhibiting greater toxicity than their larger counterparts^94^. Therefore, from a toxicity perspective, the free Ag ion, together with metallic Ag particles to a lesser degree, represents the most hazardous entities. Sulfidation or binding to sulfhydryls predominantly detoxifies silver, making it essential to comprehend the influencing elements^95^. Regulating the oxidative dissolution of Ag_2_S and the stability of organothiol complexes. Regrettably, the majority of investigations on Ag^+^ and Ag NP toxicity have overlooked the fast sulfidation that transpires in natural settings, consequently leading to a significant overestimation of toxicity^95^. A recent study in 2024 examined borate-based S49B4 bioactive glass doped with silver at mass fractions of 0.5, 1, and 3 wt%, focusing on its bioactivity, degradation, antibacterial properties, and cytocompatibility. The incorporation of silver at 1 wt% produced the most favorable results, demonstrating bactericidal efficacy of 79.8% against *E. coli* and 93.41% against *S. aureus*^96^. Correspondingly, its Lactate Dehydrogenase % closely resembles that of the negative control utilized in the study, signifying its biocompatibility. Conversely, 3 wt% silver demonstrated the highest bactericidal efficacy alongside moderate cytotoxicity. In conclusion, our research demonstrates that increased silver concentration improves the bioactivity and antibacterial properties of borosilicate bioactive glasses; nevertheless, a larger silver weight percentage in our study also raises the risk of cytotoxicity^96^. Vrček et al., (2016) assessed the toxicity of both Ag ions and citrate-coated Ag NP on human hepatoblastoma (HepG2) cells, and both materials reduced the cell viability with IC_50_ (half maximal inhibitory concentration) of Ag NP being 50 mg/L (0.5 mg/L for Ag ions)^95,97,98^. For cytotoxicity assays, Ag NPs compared to other metals release such as titanium (Ti)^98^, and Calcium (Ca)^99^, Ag NPs and Ag/Ti NPs showed a decrease in the cell viability for the two strains tested (99–50.7% for VERO and 99–45.3% for L929)^98^.

Three distinct processes that have been proposed thus far are among the factors that have been suggested to be responsible for the antibacterial activity of borate-based BG: (i) damage to the cell wall and membrane^100^; (ii) penetration and damage within the cell; and (iii) oxidative stress due to the ions released in the medium, which changes the pH of the surrounding media^38^. According to some researchers, bacterial cell membranes may be destroyed by high quantities of certain ions, such as Ag^+^ ions^32,101,102^.

### Biological evaluation of the BAgX BG samples against *E. coli* O157:H7

#### Anti-bacterial activity assay

**Fig. 13:**
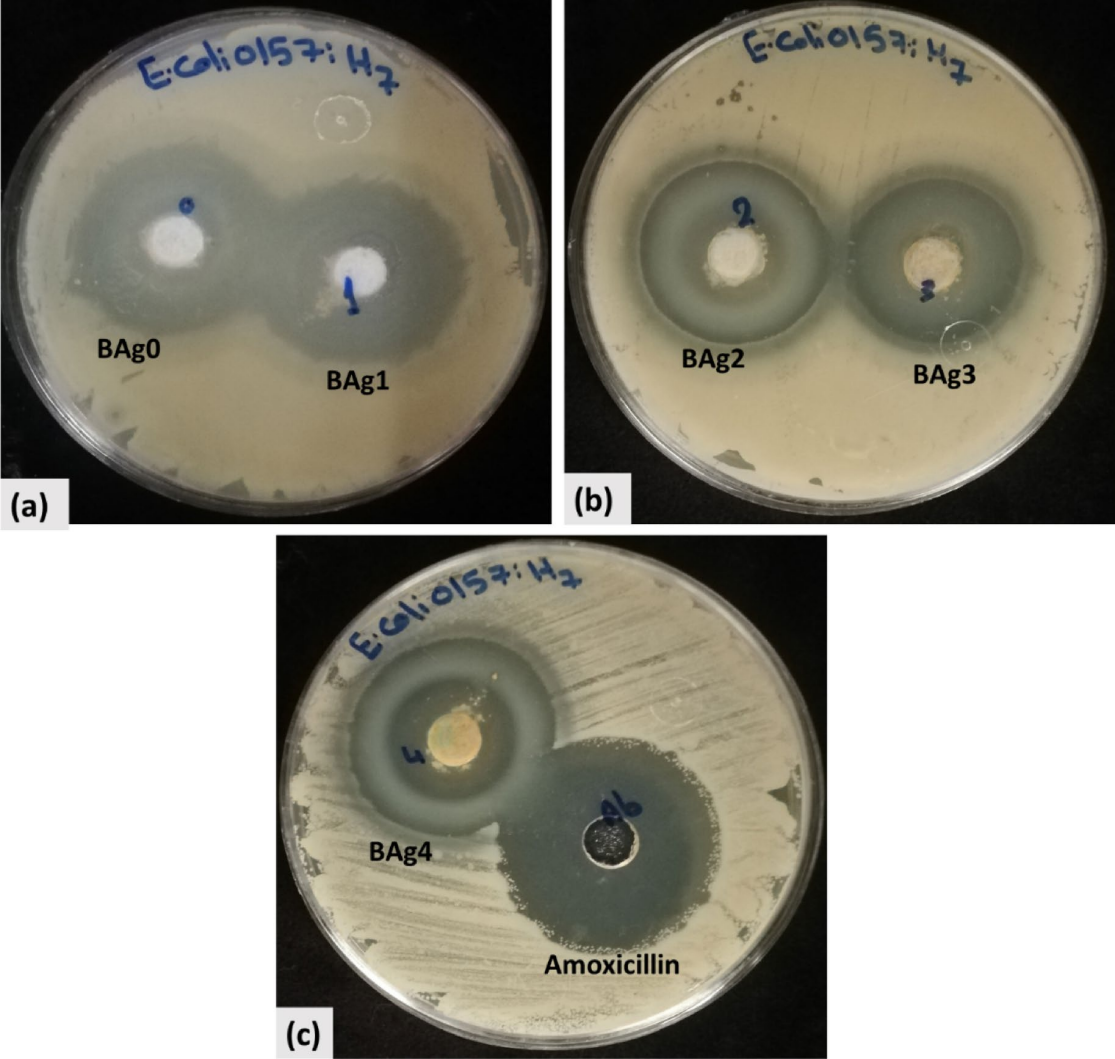
Antibacterial activity of BAgX bioglass samples against *E. coli* O157:H7 by the agar well-diffusion method on LB agar plates at 37 °C for an overnight incubation period. (**a**) BAg0 and BAg1, (**b**) BAg2 and BAg3, (**c**) BAg4 bioglass samples and amoxicillin as a positive control.

**Fig. 14:**
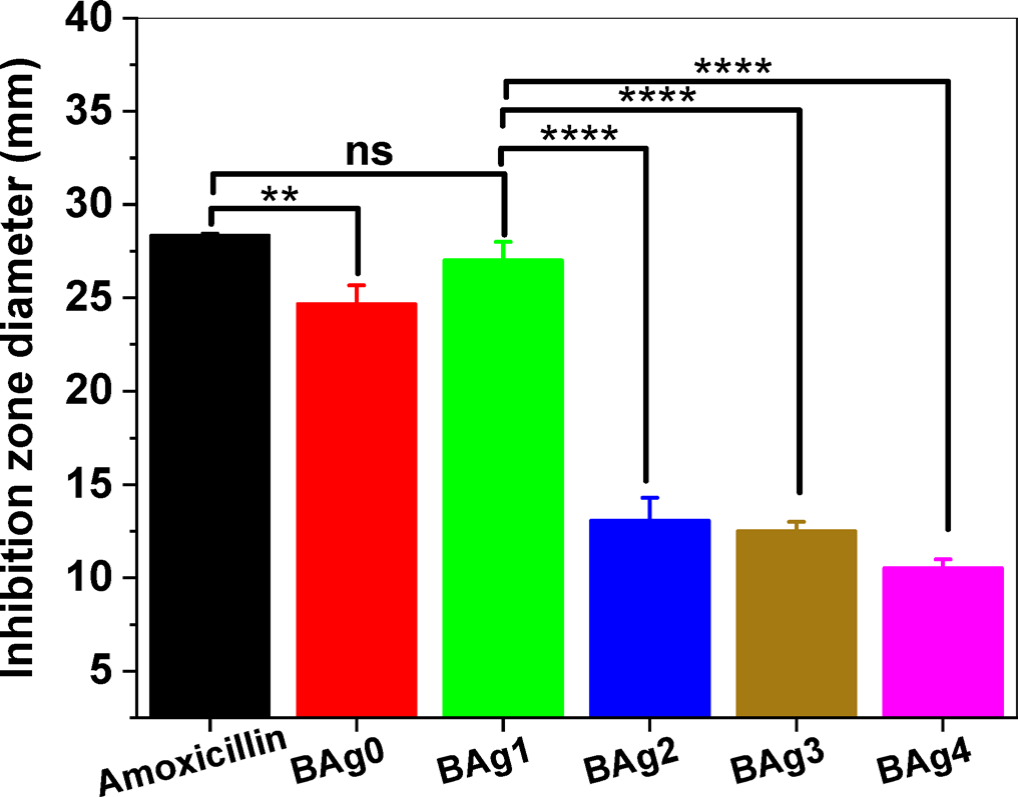
The growth inhibition zone diameter (mm) of *E. coli* O157:H7 treated with different BAg0, BAg1, BAg2, BAg3, and BAg4 bioglass samples compared to amoxicillin (positive control) by agar well-diffusion method on LB agar plates at 37 °C, (n = 3). (*p < 0.05, *p < 0.01, **p < 0.001, ***p < 0.0001 and, ****p < 0.00001).

BAgX BG samples with different concentrations of Ag_2_O were evaluated for their antibacterial activity against *E. coli* O157:H7 (CP008957). Generally, with the increase of Ag_2_O concentrations doped in borate-based BG samples, the decrease in antibacterial activity occurred against *E. coli* O157:H7 (Fig. 13). BAg1 BG sample showed the highest antibacterial activity against *E. coli* O157:H7, recording an inhibition zone of 27 mm, followed by BAg0 parent BG, recording an inhibition zone of 24.66 mm compared to amoxicillin antibiotic, as positive control as illustrated in Table 7. On the other hand, BAg2, BAg3, and BAg4 BG samples showed a reduced antibacterial effect, recording inhibition zones 13.1, 12.5, and 10.5 mm, respectively (Fig. 14). Generally comparing to the positive control, BAg0 and BAg1 BG samples exhibited a highly antibacterial effect with an efficacy of 87.05 and 95.31%, respectively. On the other hand, BAg2, BAg3, and BAg4 BG samples exhibited a reduced antibacterial effect with efficacy of 46.42, 44.12, and 37.06%, respectively.

**Table 7:** Inhibition zone diameters (mm) and growth inhibition percentages (%) of BAgX BG samples (BAg0, BAg1, BAg2, BAg3, and BAg4) against *E. coli* O157:H7 compared to the inhibition zone of amoxicillin antibiotic as positive control.

Samples		Inhibition zone diameter (mm)	Growth inhibition (%)
BAgX	BAg0	24.66 ± 1.0	87.05
	BAg1	27.00 ± 1.0	95.31
	BAg2	13.10 ± 1.08	46.24
	BAg3	12.50 ± 0.5	44.12
	BAg4	10.50 ± 0.5	37.06
Positive Control	Amoxicillin	28 .33 ± 0.1	100.00

Each value is Mean ± SD (n = 3).

The inhibition zone diameters (in mm) of Amoxicillin (positive control) and BAg0, BAg1, BAg2, BAg3, and BAG4 are shown by the bar chart (Fig. 14). With no appreciable variation between them, BAG0 and BAG1 displayed identical inhibitory zones. BAg1 exhibited the most potent antimicrobial activity among the BG samples, with an inhibition zone that was considerably larger than that of BAg2, BAg3, and BAg4 (*p* < 0.0001). With a p value of 0.0001 BAg2 displayed noticeably higher activity than BAg3 and BAg4. The most effective antibacterial agent, according to the results, is Amoxicillin; among the BG samples, BAg1 shows great promise. From BAg1 to BAG4, an apparent decrease in antibacterial efficiency is observed shown by decreasing inhibition zones and highly significant findings.

Capela et al. studied the modification of the glass (SiO_2_–CaO–P_2_O_5_–MgO) system through the incorporation of 1 and 2 mol% Ag_2_O. They stated that the antibacterial efficacy of silver may be compromised in body fluids containing species such as Cl^˗^ or proteins that can complex with ionic silver. This is attributed to the formation of insoluble silver salts, which reduce the concentration of silver ions in solution^33^.

Mulligan and his colleagues revealed that silver-doped phosphate-based bioactive glasses exhibit promising antibacterial capabilities against planktonic microorganisms. The antibacterial efficacy of various amounts of silver doping (1, 5, 10, and 15 mol%) was evaluated. The findings indicated that the presence of silver resulted in a decrease in colony-forming units (CFU) of bacteria. Almost complete eradication of bacteria was seen after 24 h for 5% Ag, while higher concentrations demonstrated even better suppression; the gradual increase of the CFU curve was ascribed to a reduction in Ag diffusion during biofilm penetration^103^.

Furthermore, a bioactive glass scaffold composed of 70S30C (70SiO_2_-30CaO) with the incorporation of 1 mol% Ag_2_O was synthesized via the sol–gel method. This scaffold demonstrated the release of 0.95 μg/ml of Ag^+^ ions over a period of 23 h, exhibiting a bactericidal effect against cultures of *E. coli, P. aeruginosa, and S. aureus*^104^. A comparative analysis of the antibacterial properties of a sol–gel derived silver-doped 64SiO_2_-26CaO-10P_2_O_5_ (mol%) bioactive glass and its undoped equivalent against *E. coli* species revealed that the silver-free bioactive glass exhibited neither bacteriostatic nor bactericidal effects. In contrast, the bioactive glass doped with 5 mol% Ag_2_O demonstrated a killing efficiency exceeding 99% against *E. coli*^70^. In pertinent investigations, 4 and 8 wt% Ag_2_O were incorporated into the 62.3SiO_2_–28.9CaO-8.6P_2_O_5_ (wt%) sol–gel derived bioactive glass to examine and juxtapose their antibacterial properties with those of the undoped bioactive glass in relation to *E. coli* DH5α ampicillin-resistant and Streptococcus mutans (*S. mutans*)^105^.

Ag ions interfere with bacterial cells’ metabolic processes by deactivating proteins and preventing DNA replication^106^. By looking at the test findings, it was easy to see that the sample without Ag_2_O did not exhibit an inhibitory zone, proving that the samples’ antibacterial activity is caused by Ag ions dissolving in the agar media^107^.

#### MIC and MBC of BAgX BG samples

The MIC value was defined as the lowest concentration of BAgX BG samples inhibiting the growth of *E. coli* O157:H7. By measuring the growth of *E. coli* O157:H7 at a specific concentration of BAgX BG samples, the minimum inhibitory concentration (MIC) assay was used to assess the antibacterial potency of BAgX BG samples (Fig. S 4). Generally, BAg1, BAg2, BAg3, and BAg4 BG samples exhibited their antibacterial effect even at the lowest concentration (5 mg/mL), which reflects the high activity as an antibacterial agent.

Compared to untreated bacterial culture as a negative control, BAg1, BAg2, BAg3, and BAg4 were the most effective candidates, showing the best MIC value of 5 mg/mL. On the other hand, the BAg0 parent BG sample displayed a MIC value of 50 mg/mL and, subsequently, the BAg0 parent BG had the lowest antibacterial activity against *E. coli* O157:H7. The MBC value is the lowest concentration of BAgX BG samples required to kill *E. coli* O157:H7. Compared to the negative control, BAg1, BAg2, BAg3, and BAg4 treatments showed the same MBC value of 50 mg/mL (Fig. S5). On the other hand, the MBC value of the BAg0 parent BG sample was not achieved until 50 mg/mL as depicted in Table 8. In vitro, the scaffolds doped with 0.5 and 1.0 wt% Ag_2_O exhibited a bacterial inhibition of.90% against *S. aureus* and *E. coli*^47^. Further, the scaffolds that were doped with 1.0 wt% were treated for three days in SBF at 37 °C. Ag_2_O stopped 66% and 71% of *S. aureus* and *E. coli*, respectively, from growing, which suggests that they could stop germs from growing for a longer time^47^.

**Table 8:** MIC and MBC values (ppm) of BAgX BG samples (BAg0, BAg1, BAg2, BAg3, and BAg4) against *E. coli* O157:H7.

BAgX BG samples	MIC (g/L)	MBC (g/L)
BAg0	50.0	> 50.0
BAg1	5.0	50.0
BAg2	5.0	50.0
BAg3	5.0	50.0
BAg4	5.0	50.0

#### Detection of Ag-doped borate BG samples-resistant variants

**Fig. 15:**
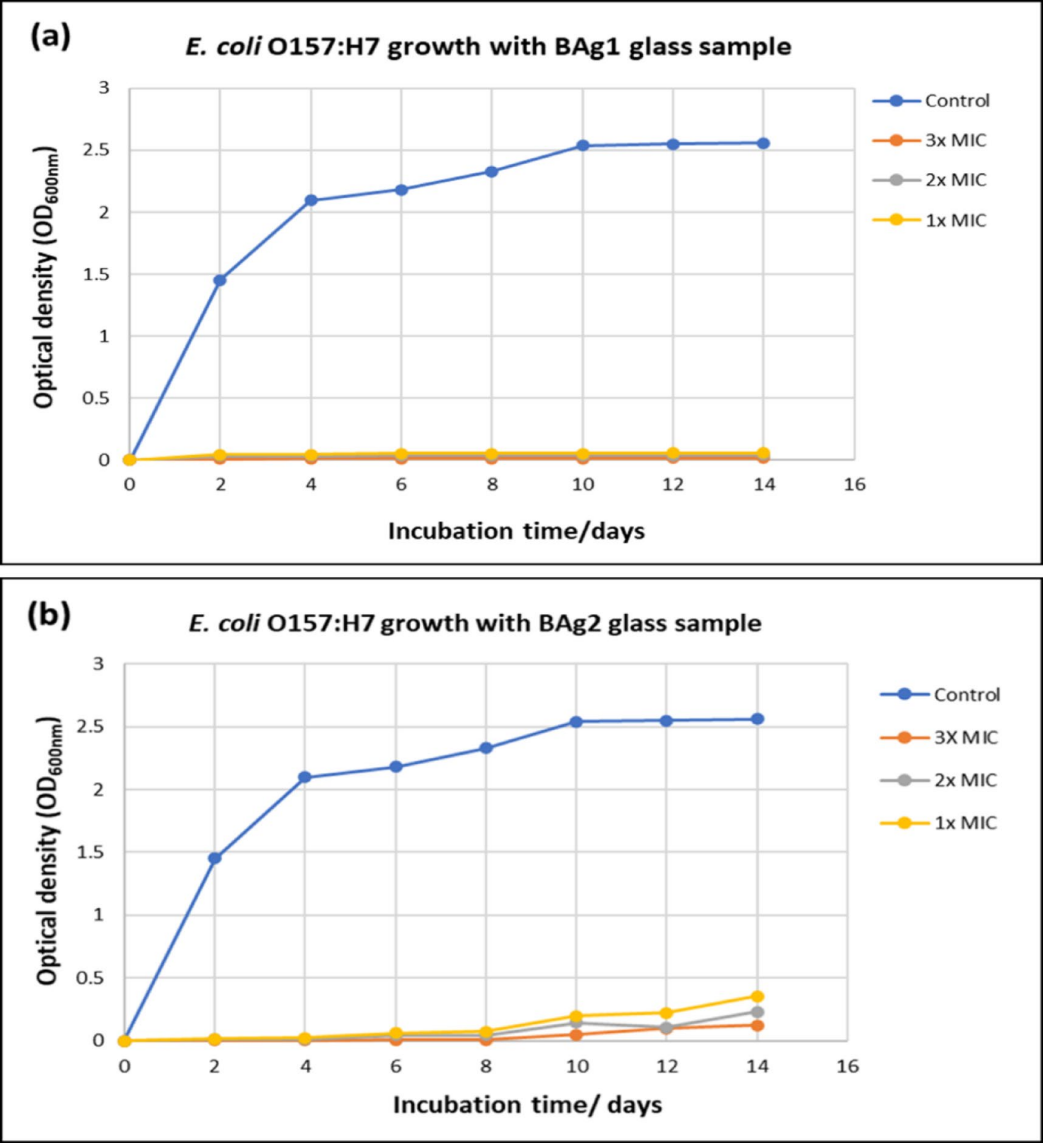

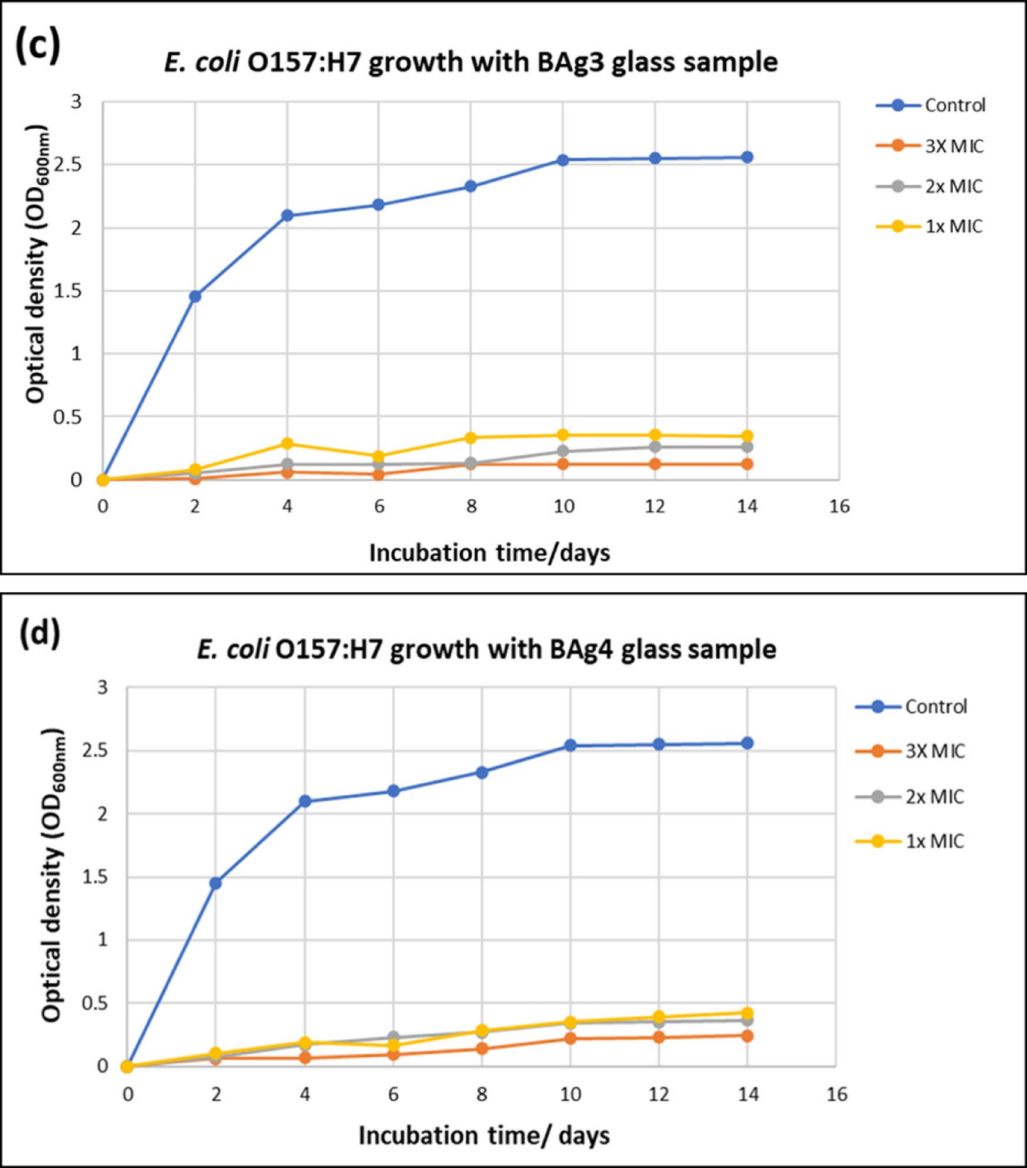
Resistance development test of *E. coli* O157:H7 after treatments with different folds of MBC 1, 2, and 3X of BAg1 (**a**), BAg2 (**b**), BAg3 (**c**), and (**d**) BAg4 bioglass samples at 37 °C and 150 rpm for 14-day incubation period compared to untreated bacterial culture as a negative control.

Based on the MBC results, the BAg0 parent BG, free of Ag_2_O, did not reach the estimated MBC value of 50 mg/mL, therefore, the resistant development test wasn’t applied to the BAg0 parent BG sample. Figure 15 represents the development of spontaneous resistance conducted for a 14-day incubation period only for *E. coli* O157:H7 treated with BAg1, BAg2, BAg3, and BAg4 BG samples at different doses 1, 2, and 3 times MBC, respectively. It can be noticed that the bacterial *E. coli* O157:H7 treated with BAg1 BG sample shows the most resistant investigated sample among the investigated BG samples as shown in Fig. 15a. Conversely, the BG samples (BAg2, BAg3, and BAg4) represented some resistant behaviour compared to BAg1 BG (Fig. 15b–d). Furthermore, the digital photographs of the plates for untreated bacterial culture (negative control), BAg1, BAg2, BAg3, and BAg4 BG samples could effectively prevent the growth of *E. coli* O157:H7 were illustrated in Fig. S6. Therefore, the bacterial *E. coli* O157:H7 did not show any resistance at higher concentrations of 1, 2, and 3X MBC, respectively. It is reported that *E. coli* growth is inhibited by Ag-BG concentrations of 0.05–0.20 mg/ml, while complete bactericidal effects are achieved within hours at 10 mg/ml^108^. Higher silver doping (up to 7.5 mol%) enhances antibacterial activity without compromising biocompatibility. Nevertheless, surface reactivity may be diminished by excessive influence (> 7.5 mol%)^108^.

#### SEM analysis of *E. coli* O157:H7

**Fig. 16:**
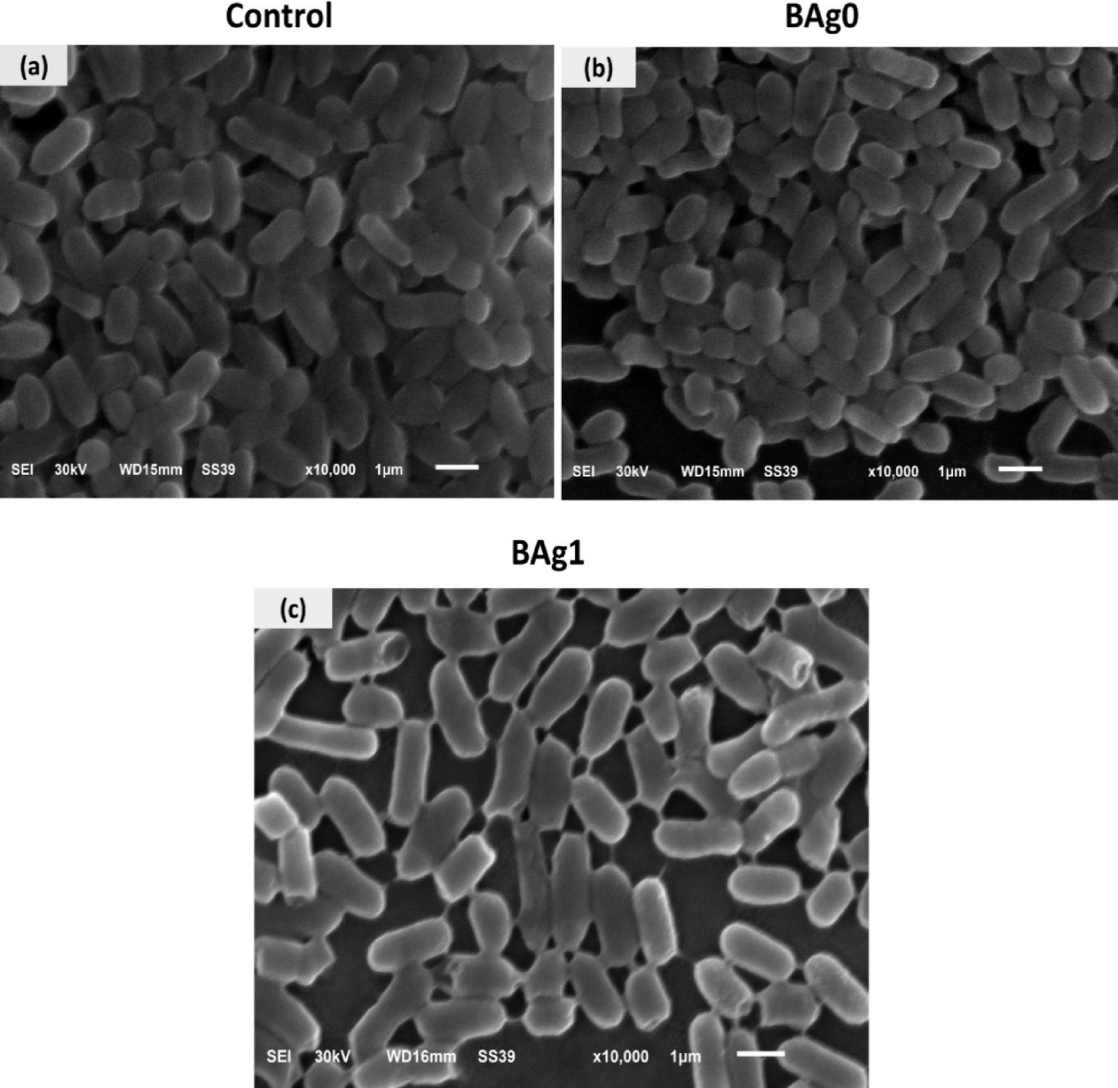
SEM micrographs of *E. coli* O157:H7 before treatments (**a**) and after treatments with BAg0 (**b**) and BAg1 bioglass samples (**c**).

The SEM micrographs of *E. coli* O157:H7 bacterial cells are depicted in Fig. 16. The SEM image of the untreated *E. coli* is depicted in Fig. 16a. The untreated cells are characterized by normal morphology, short rods, smooth, and intact cell walls. To examine the effect of the parent BAg0 BG sample on the *E. coli* cells, the SEM image of the sample treated with sub-MIC concentrations is represented in Fig. 16b. It can be noticed that few bacterial cells are abnormal in shape, and few other cells are more significant than normal cells. In contrast, for the BAg1 BG sample, the morphological deterioration in the bacterial cell is greater than in the BAg0 parent BG sample (Fig. 16c).

Moreover, these morphological changes of many bacterial cells like partial lysis or trimming, irregular shape, swollen, crumpled, elongated, and collapsed were exposed when compared to untreated cells. Also, there are noticeable changes in the sizes of the bacterial cells treated with the BAg1 BG sample. Additionally, the size of some cells is larger (> 2 µm) than normal cell size (around 1.5 µm), whereas other cells are smaller in size. Furthermore, there is some sort of aggregation or connection between bacterial cells through several bridges, connections, or conjugation tubes (Fig. 16c).

**Fig. 17:**
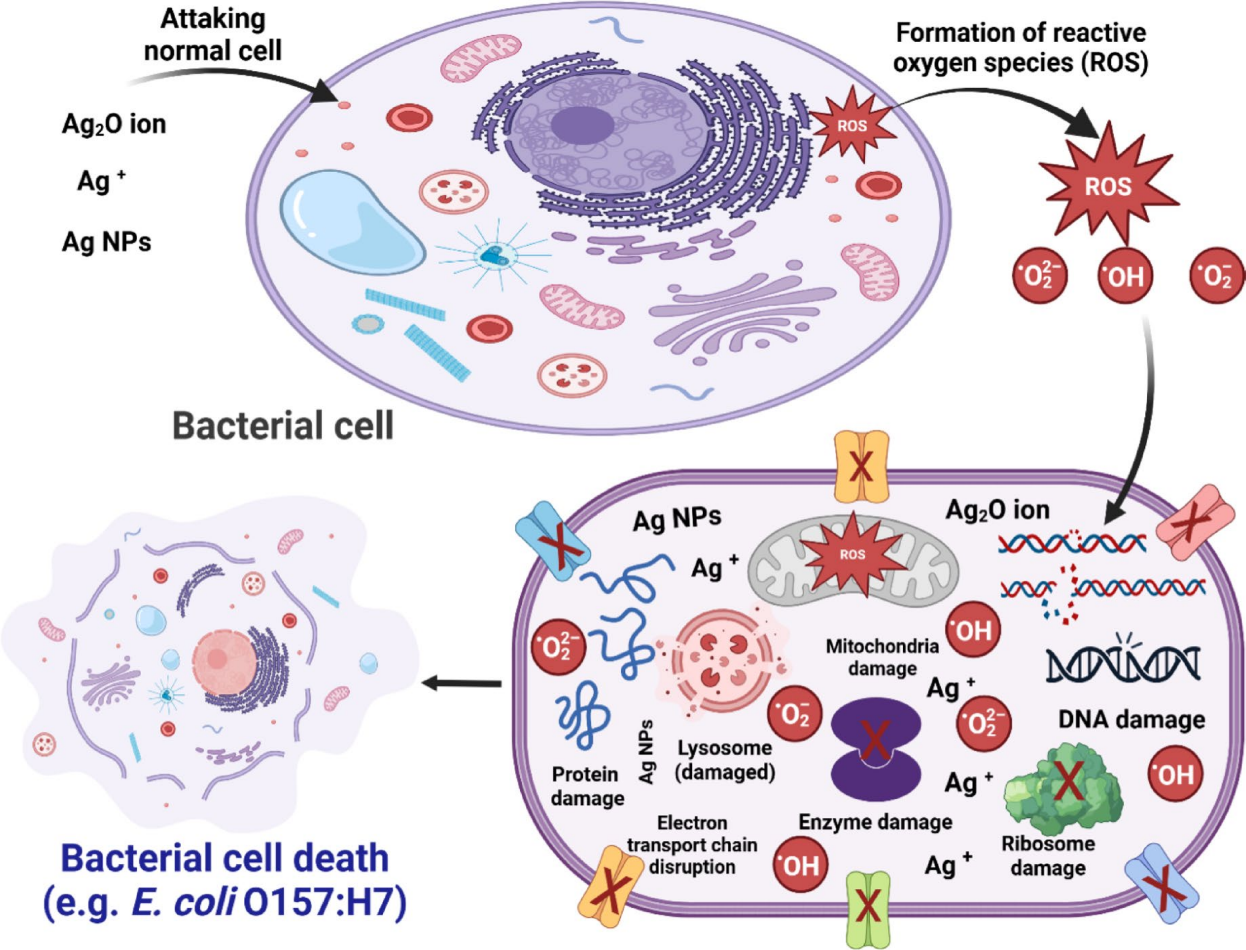
The proposed bacterial cell (e.g. *E. coil O157:H7*) death due to the Ag multi-forms (i.e., ions, oxides, and nanoparticles) in the bioglass.

Silver ions may attack bacteria in several ways, so they are quite successful against many harmful microbes. To elucidate the role of Ag^+^ ions in the *E. coli* O157:H7 bacterial cell death, many assumptions were reported to discuss its role^109–111^. Figure 17 represents a proposed mechanism of the role of Ag_2_O, Ag^+^ ions, and/or the metallic Ag^0^ NPs of the *E. coli* O157:H7 bacterial cell death. Silver ions may attach to the bacterial cell walls and membranes, and they weaken and increase the permeability of these structures^109^. Eventually, this damage can cause the bacterial cell to disintegrate and die. The bacteria could survive using certain proteins and enzymes, whereas Ag^+^ ions attach to these proteins, particularly those with sulphur groups, therefore preventing their normal operation. Without these essential proteins, bacteria cannot perform necessary functions including breathing, which causes their death. Furthermore, it is possible that the inhibitory action of borate-based BG results from the production of reactive oxygen species (ROS) and the release of boron, calcium, phosphate, and Ag^+^ ions, which alters the bacterial membrane potential and raises the osmotic pressure^110^.

On the other hand, oxidative stress can be brought on by reactive oxygen species such as hydrogen peroxide (H_2_O_2_), hydroxyl radicals (-OH), superoxide radicals (O_2_^-^), and singlet oxygen (O_2_). For instance, H_2_O_2_ may penetrate the intracellular space of the bacteria and break their cell membrane, which would then damage the bacteria’s proteins, DNA and lipids^111,112^. These microorganisms are ultimately destroyed as a result of the oxidative stress they experience. Silver ions disrupt the DNA of a bacterial cell once it is inside, therefore stopping its division and replication^113^. In the absence of the capacity to reproduce, the microbes ultimately die. The electron transport system is how bacteria establish energy. By disturbing this system, silver ions cause a loss of energy generation (ATP depletion) and finally bacterial death^112,114^. Unlike conventional antibiotics, which often target only one particular function, Ag ions have one of the main benefits in that they kill bacteria in several ways, thereby making it far more difficult for bacteria to acquire resistance. Silver is thus widely utilised in medical applications, wound dressings, medical device coatings, and even water purification systems to help avoid infections^114^.

## Conclusion

Countries worldwide aim to purify wastewater to conserve freshwater due to its limited availability. Effective wastewater treatment and reuse are advised. Therefore, various investigations and research were done to reach this purpose. BG was introduced as an advanced SWW purification process. The melting-quenching procedure prepared BAgX BG samples. The composition was (65-X) B_2_O_3_-20Na_2_O-10CaO-5P_2_O5-XAg_2_O (X = 0, 1, 2, 3, and 4 mol%). Amorphous phases were found in both un-doped and Ag-doped borate BG powders. No grain boundaries were visible on the surface in SEM pictures. The DLS results show that the parent BAg0 BG sample had an average particle size of 738 ± 151 nm, while the BAg1 sample had 380 ± 65 nm. Using the SPC method, BAg1, BAg2, BAg3, and BAg4 BG samples inhibited bacterial community and coliform bacteria 99.9% and 100%, respectively. Unlike the positive control calcium hypochlorite (Ca (ClO)_2_), the parent BAg0 BG sample did not limit bacterial growth. Based on presumptive analysis, BAg1 was the most effective treatment for sewage wastewater samples, with coliform bacterial load ≤ 5000 MPN/100 mL. BAg2, BAg3, and BAg4 had coliform bacterial loads of 180, 680, 920, and 1400 MPN/100 mL, respectively. Additionally, the confirmation test for E. coli showed that BAg1, BAg2, BAg3, and BAg4 BG samples were free of *E. coli*, while BAg0 BG samples included E. coli as a fecal contamination signal. The increase in Ag_2_O concentration doped in borate BG samples and the decrease in Ag^+^ ions produced were likely to diminish the antibacterial activity of borate-based BG doped with Ag_2_O. BAg0 and BAg1 BG samples had 87.05 and 95.31% effectiveness against E. coli O157:H7, respectively, with MIC values of 5 and 50 mg/mL, compared to amoxicillin. However, BAg2, BAg3, and BAg4 BG samples had low antibacterial activity of 46.42, 44.12, and 37.06% against E. coli O157:H7 with MIC values of 5 mg/mL. BAg0 parent BG sample had no MBC value against *E. coli* O157:H7, whereas BAg1, BAg2, BAg3, and BAg4 demonstrated bactericidal activity at 50 mg/mL. E. coli O157:H7 did not develop resistance to BAg1, BAg2, BAg3, and BAg4 BG samples over the 14-day incubation. SEM micrographs showed that BAg1-treated *E. coli* O157:H7 cells had more morphological degradation than BAg0-treated cells. Finally, the BAg1 BG sample purifies sewage effluent from coliform bacteria efficiently. The antibacterial effect of borate-based BG against fecal coliform bacteria and pathogenic bacteria in sewage wastewater samples, especially *E. coli* O157:H7, a model water-borne pathogen, was enhanced by doping Ag_2_O at 1 mol%. For water waste purification from fecal coliform bacteria, borate-based BG doped with silver is highly recommended.

## Electronic supplementary material

Below is the link to the electronic supplementary material.

Supplementary Material 1

## Data Availability

The data will be available from the corresponding authors for a reasonable reason.
