# Correction: Removal of *E. coli* O157:H7 coliform bacteria from sewage wastewater using silver doped borate bioglass

**DOI:** 10.1038/s41598-025-22905-3

**Published:** 2025-10-07

**Authors:** Bassant I. Elsaba, Ashraf Elsayed, Yasser S. Rammah, Hosam Salaheldin

**Affiliations:** 1 Botany Department, Faculty of Science, Mansoura University, Mansoura, 35516 Egypt; 2 Department of Physics, Faculty of Science, Menoufia University, Shebin El-Koom, 32511 Menoufia Egypt; 3 Biophysics Research Group, Physics Department, Faculty of Science, Mansoura University, Mansoura, 35516 Egypt

Correction to: *Scientific Reports* 10.1038/s41598-025-11844-8, published online 26 July 2025

The original version of this Article contained an error in the spelling of the author Ashraf Elsayed which was incorrectly given as Ashraf Elsayad.

The original Article has been corrected.

