# Supplementary material for: Removal of E. coli O157:H7 coliform bacteria from sewage wastewater using silver doped borate bioglass
Source: Sci Rep. 2025 Jul 26;15:27212. doi: 10.1038/s41598-025-11844-8 (PMC12297699; doi:10.1038/s41598-025-11844-8)
Supplement: Supplementary file 1 — Supplementary Material 1 [file 41598_2025_11844_MOESM1_ESM.docx]

**Supplementary file**

**S1.** MPN index and 95% confidence limits for various combinations of positive results when five tubes are used per dilution (5 mL, 1 mL, 0.1 mL)**.**

**
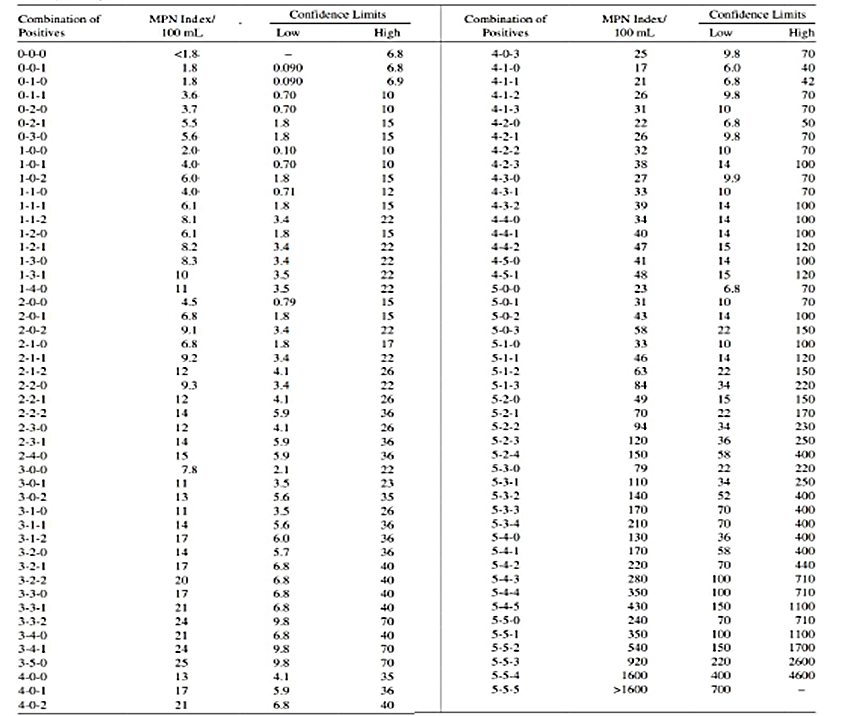
**

**
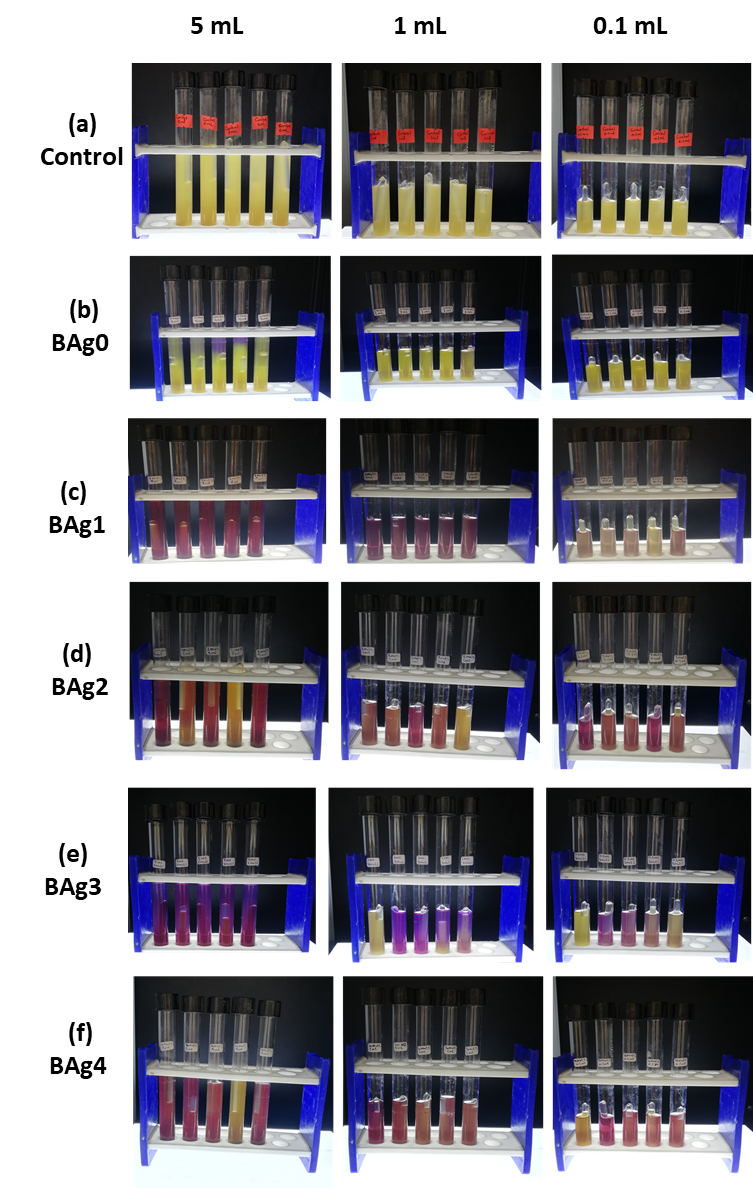
**

**S2.** Presumptive test for the presence of coliforms in sewage wastewater samples **after 24 h** treatments using; (a) untreated or control sample, (b) BAg0, (c) BAg1, (d) BAg2, (e) BAg3, and (f) BAg4 glass samples on MacConkey broth purple media. Both of yellow color and gas formation indicate the presence of coliforms in sewage wastewater samples. Purple color and/or no gas formation is a negative result.


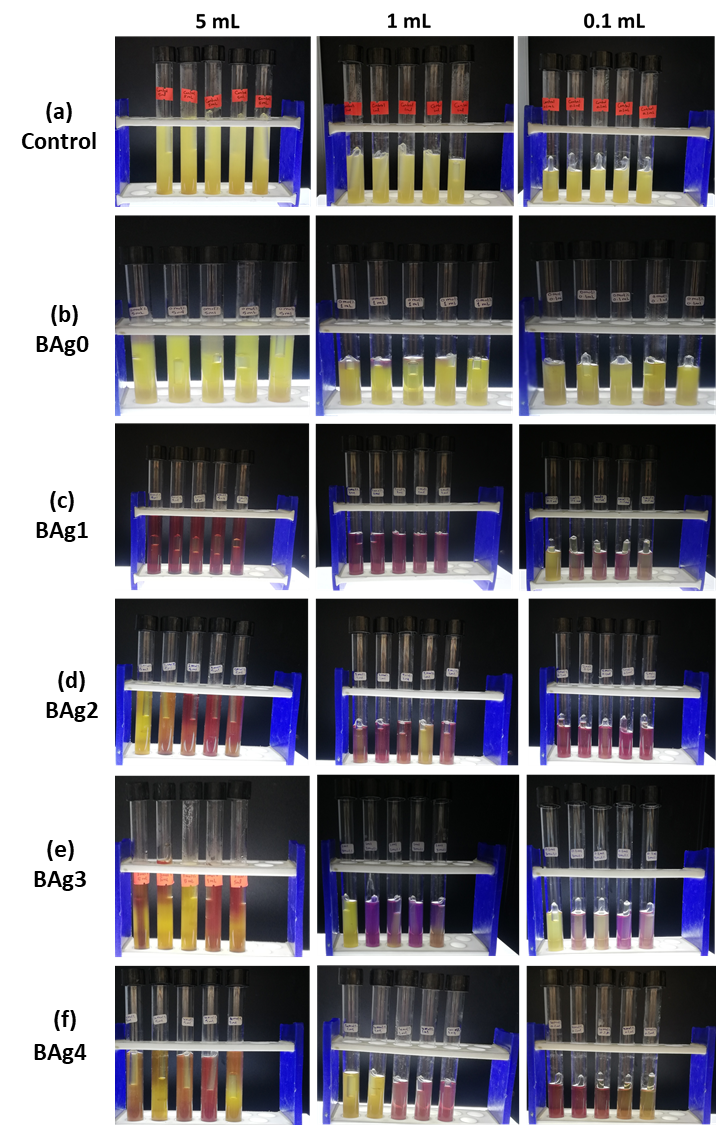


**S3.** Presumptive test for the presence of coliforms in sewage wastewater samples **after 48 h** treatments using; (a) untreated or control sample, (b) BAg0, (c) BAg1, (d) BAg2, (e) BAg3, and (f) BAg4 glass samples on MacConkey broth purple media. Both of yellow color and gas formation indicate the presence of coliforms in sewage wastewater samples. Purple color and/or no gas formation are negative results.


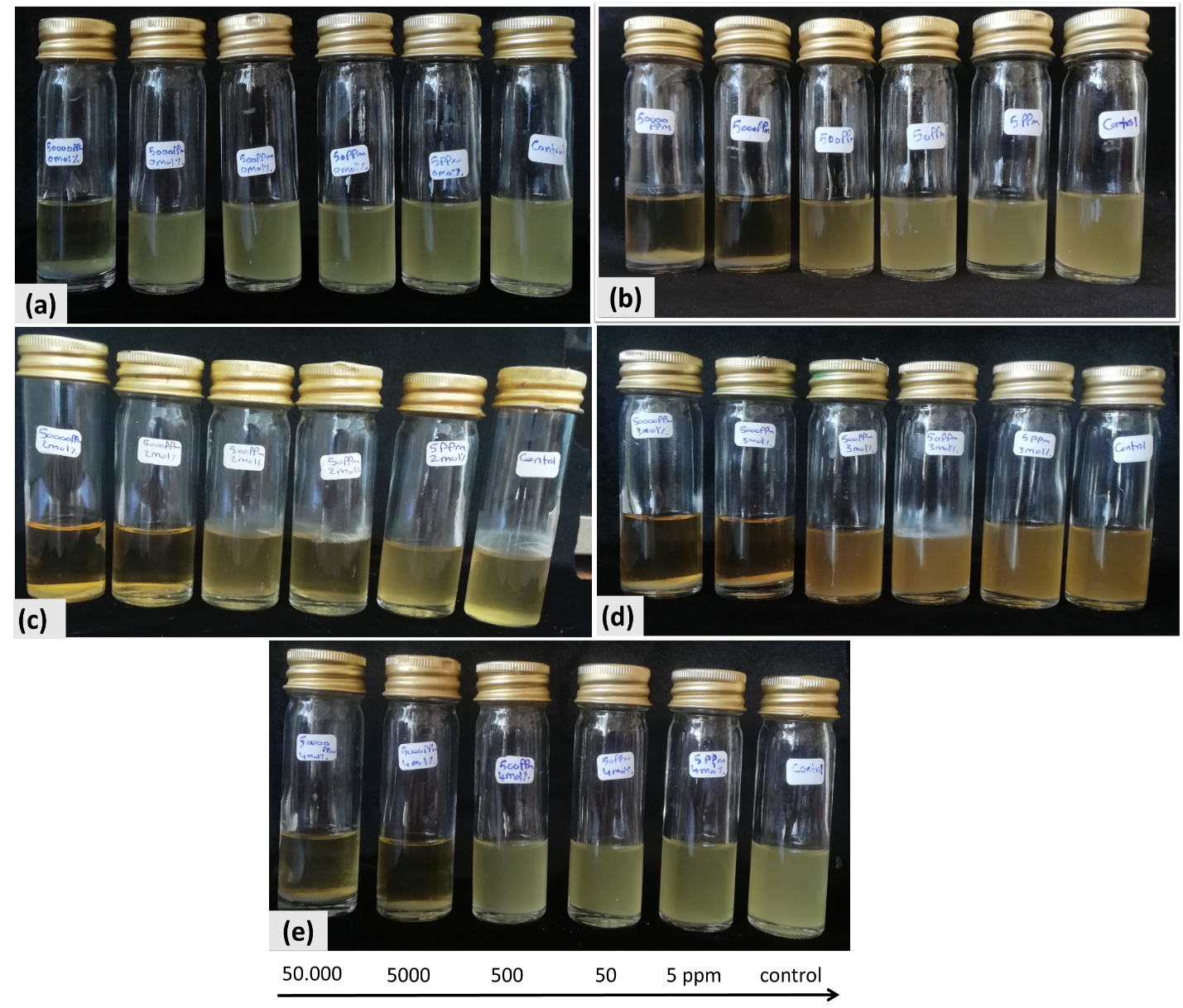


**S4.** MIC determination after different treatments of *E. coli* O157:H7 with different concentrations (5, 50, 500, 5000, and 50.000 ppm) of BAgX glass samples in LB broth at 37 °C, 150 rpm, for an overnight incubation period compared to untreated bacterial culture as a negative control. (a) BAg0 parent glass, (b) BAg1, (c) BAg2, (d) BAg3, and (e) BAg4 glass samples.


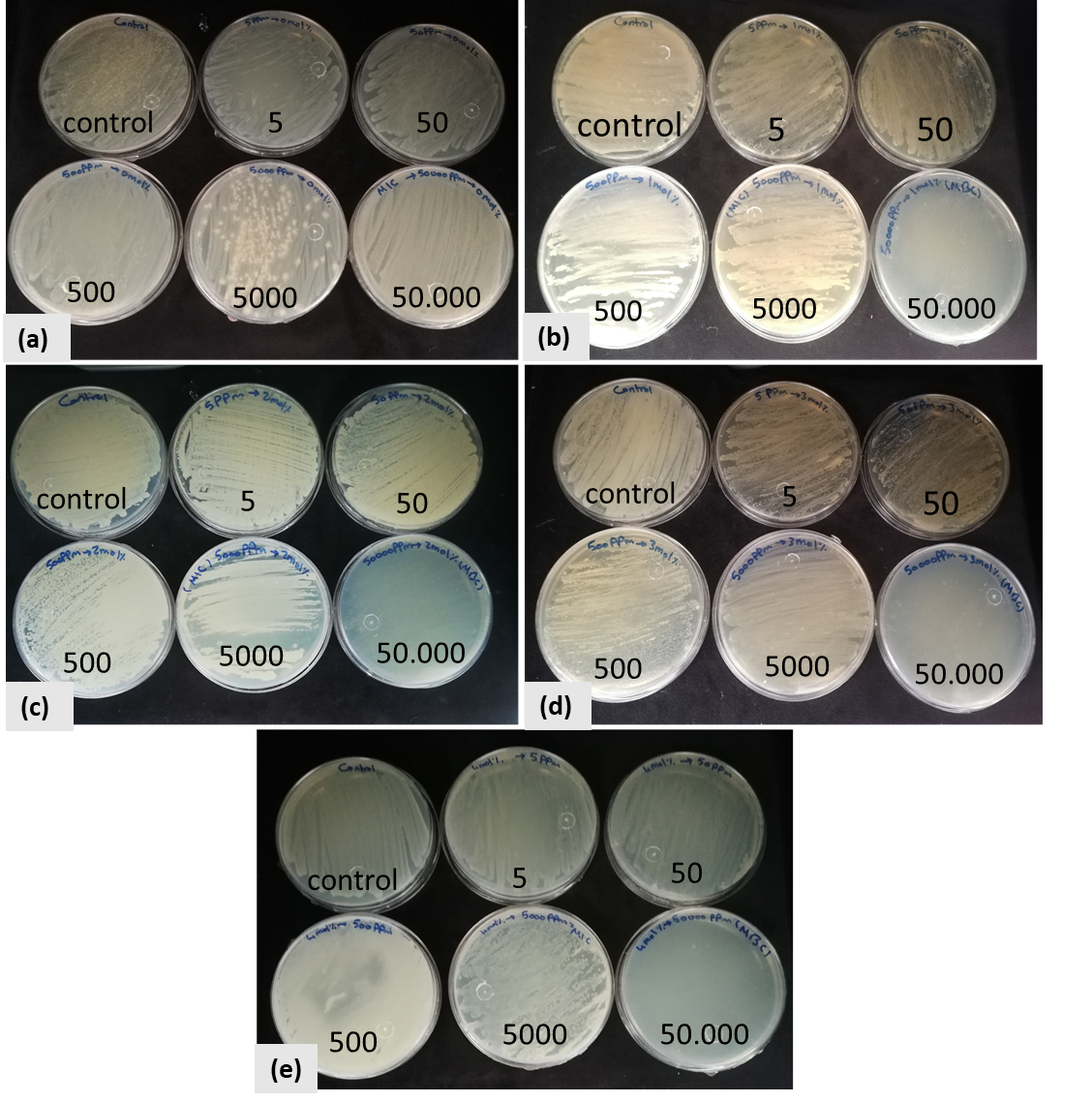


**S5.** MBC determination after subculturing *E. coli* O157:H7 treated with different concentrations (5, 50, 500, 5000, and 50.000 ppm) of BAgX glass samples on LB agar plates at 37 °C for the overnight incubation period compared to the negative control. (a) BAg0 parent glass, (b) BAg1, (c) BAg2, (d) BAg3, and (e) BAg4 glass samples.


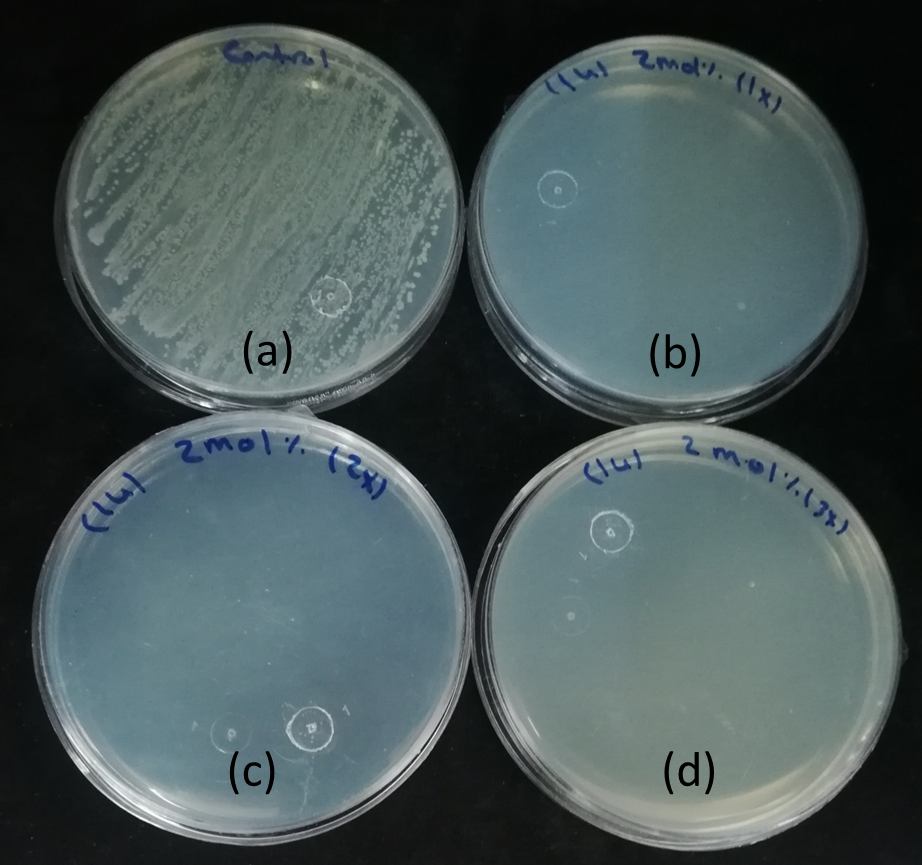


**S6.** Representative plates from the resistance development test of *E. coli* O157:H7 after treatments with different folds of MBC 1, 2, and 3X of BAg2 glass sample at 37 °C and 150 rpm for a 14-day incubation period compared to the negative control. Where (a) control; (b) 1x MIC; (c) 2x MIC; (d) 3x MIC of BAg2 glass sample.
